# Lisdexamfetamine in the treatment of methamphetamine dependence: A randomised, placebo‐controlled trial

**DOI:** 10.1111/add.16730

**Published:** 2024-12-19

**Authors:** Nadine Ezard, Brendan Clifford, Krista J. Siefried, Robert Ali, Adrian Dunlop, Rebecca McKetin, Raimondo Bruno, Andrew Carr, James Ward, Michael Farrell, Robert Graham, Paul Haber, Dan Lubman, Mark W. Donoghoe, Nick Olsen, Amanda Baker, Michelle Hall, Shalini Arunogiri, Nicholas Lintzeris, LiMA Investigator Group, Anthony Gill, Craig Rodgers, Mark Montebello, Will Liaw, Zhixin Liu

**Affiliations:** ^1^ National Centre for Clinical Research on Emerging Drugs University of New South Wales Sydney Australia; ^2^ Alcohol and Drug Service, St Vincent's Hospital Sydney Sydney Australia; ^3^ National Drug and Alcohol Research Centre University of New South Wales Sydney Australia; ^4^ Drug and Alcohol Clinical Research and Improvement Network, NSW Health Sydney Australia; ^5^ Faculty of Health and Medical Sciences University of Adelaide Adelaide Australia; ^6^ University of Newcastle Newcastle Australia; ^7^ Hunter New England Local Health District Newcastle Australia; ^8^ School of Psychological Sciences University of Tasmania Hobart Australia; ^9^ Applied Medical Research, St Vincent's Hospital Sydney Australia; ^10^ Poche Centre for Indigenous Health University of Queensland Queensland Australia; ^11^ Western Sydney Local Health District Sydney Australia; ^12^ Sydney Local Health District Sydney Australia; ^13^ Discipline of Addiction Medicine University of Sydney Sydney Australia; ^14^ Turning Point, Eastern Health Melbourne Australia; ^15^ Monash Addiction Research Centre, Eastern Health Clinical School Monash University Melbourne Australia; ^16^ Clinical Research Unit University of New South Wales Sydney Australia; ^17^ Kirby Institute University of New South Wales Sydney Australia; ^18^ Mark Wainwright Analytical Centre University of New South Wales Sydney Australia; ^19^ Drug and Alcohol Services, South Eastern Sydney Local Health District Sydney Australia; ^20^ Northern Sydney Local Health District Sydney Australia; ^21^ Drug and Alcohol Service of South Australia Adelaide Australia

**Keywords:** Clinical trial, lisdexamfetamine, methamphetamine, methamphetamine dependence, methamphetamine use disorder, randomized controlled trial, non‐abstinence outcomes, stimulants

## Abstract

**Aims:**

This study tested the efficacy and safety of a 12‐week course of lisdexamfetamine in reducing methamphetamine use, an outcome which is associated with improvements in health and wellbeing, in people dependent on methamphetamine.

**Design, setting and participants:**

This study was a randomised double‐blind placebo‐controlled trial conducted in six specialist outpatient clinics in Adelaide, Melbourne, Newcastle and Sydney, Australia (2018–2021). Participants were164 adults with methamphetamine dependence, reporting at least 14 use days out of the previous 28 days (62% male, 38% female, < 1% other; mean age 39 years).

**Interventions:**

Participants were randomly allocated 1:1 to a 15‐week regimen of lisdexamfetamine (1‐week induction to 250 mg, 12‐week maintenance regimen, 2‐week reduction; *n* = 80) or matched placebo (*n* = 84), followed‐up to Week 19.

**Measurements:**

The primary efficacy measure was past 28‐day methamphetamine use at Week 13. Safety was assessed by adverse event rates. Secondary measures included methamphetamine use during the 12‐week treatment period and treatment satisfaction.

**Findings:**

Nine randomized participants did not start treatment (five were allocated to lisdexamfetamine and four allocated to placebo) and were excluded from the analyses. Fifty‐seven per cent of participants were retained on study medication to primary end‐point. There was only weak evidence of a lisdexamfetamine benefit at 13 weeks [adjusted difference in days of methamphetamine use = 2.2, 95% confidence interval (CI) = –0.5 to 5.0; *P* = 0.49]. However, throughout the whole 12‐week treatment maintenance phase, the lisdexamfetamine group had fewer days of methamphetamine use in total (difference = 8.8, 95% CI = 2.7–15.0; *P* = 0.005). The lisdexamfetamine group reported greater self‐reported treatment effectiveness [odds ratio (OR) = 2.89, 95% CI = 1.67–5.02; *P* < 0.001] and treatment satisfaction (OR = 3.80, 95% CI = 1.93–7.47; *P* < 0.001). Adverse events with lisdexamfetamine included nausea. Serious adverse events occurred in four (5%) of participants who received lisdexamfetamine.

**Conclusions:**

Lisdexamfetamine appears to reduce methamphetamine use over a 12‐week treatment period, although there is only weak evidence that reduced use is maintained during the last 4 weeks.

## INTRODUCTION

An estimated 36 million people world‐wide use amphetamine‐type substances, including methamphetamine [1]. Methamphetamine use is associated with physical, psychological and social harms, as well as increased mortality [2]. Current evidence provides support for psychosocial interventions, such as cognitive behavioural therapy (CBT), in improving treatment retention [3]. The strongest efficacy evidence is from the United States, supporting contingency management with abstinence‐based reinforcement [3]. Effectiveness of psychosocial interventions is weak among those using very frequently (at least half of the days in a month) [4]. In contrast to other substance use disorders, there are no pharmacotherapies for this condition [5].

Lisdexamfetamine presents a promising candidate agonist‐like pharmacotherapy for methamphetamine dependence. As a prodrug of dexamphetamine, it has a lower non‐medical use potential than dexamphetamine as it requires hydrolysis into active dexamphetamine [6], has no differential subjective effect if injected [7] and has a slower onset and longer duration of action [8], avoiding the potential positive reinforcement associated with immediate release stimulant medications [7]. Higher doses may be required for methamphetamine dependence [9] than for Attention Deficit Hyperactivity Disorder (ADHD) or Binge Eating Disorder, where up to 70 mg is indicated [10]. One hundred mg lisdexamfetamine is equivalent to 40 mg dexamphetamine base [11]; doses of 60–110 mg dexamphetamine have been trialled for amphetamine dependence [12, 13]. Our group's pilot work informing this study demonstrated that doses starting at 100 mg and increasing to 250 mg of lisdexamfetamine are safe in this population [14]. Our more recent work on withdrawal in this population used a starting dose of 250 mg lisdexamfetamine with no safety concerns [15].

We sought to test the efficacy and safety of lisdexamfetamine in reducing methamphetamine use for people dependent on methamphetamine using at least 14 days in the previous 28. We hypothesised that participants who received a 12‐week course of 250 mg of lisdexamfetamine would report fewer days of methamphetamine use in the last 28 days of treatment compared to participants who received a placebo control.

## METHODS

### Study design

This was a randomised double‐blind placebo‐controlled Phase III fixed‐dose parallel design trial; the protocol has been previously published [16]. In brief, participants were recruited from six Australian specialist stimulant treatment clinics (Adelaide, Melbourne, Newcastle, Sydney), randomized 1:1 to oral lisdexamfetamine (250 mg daily for 12 weeks, plus 1‐week induction and 2‐week taper) or identical matched placebo, and followed after 4 weeks (Week 19). Amendments due to COVID‐19 pandemic public health restrictions are described below [17]. Ethics approval was granted by St Vincent's Hospital Sydney Human Research Ethics Committee (HREC, 2019/ETH03140). This study followed the Consolidated Standards of Reporting Trials (CONSORT) reporting guideline [18]. All participants provided written, informed consent.

### Participants

Participants were recruited through local clinics and online advertisements. Following telephone pre‐screening, consenting participants underwent assessment by a study medical officer to determine if all eligibility criteria were met, and were then randomised. Adults assessed by an addiction medicine specialist to meet ICD‐10 criteria for methamphetamine dependence [19] reporting use of methamphetamine on at least 14 of the previous 28 days, with one urinalysis positive for methamphetamine, were eligible. Exclusion criteria included known contraindications to lisdexamfetamine [10] (apart from history of stimulant dependence), current opioid agonist therapy, concurrent severe psychiatric or other medical disorder, dependent use of alcohol or other non‐prescribed substances which, in the opinion of the investigator, would interfere with participation in the study, use of another prescribed stimulant (such as methylphenidate), unavailability for follow‐up or undergoing child protection, court or work‐mandated drug testing.

### Interventions

Participants received daily lisdexamfetamine (1‐week induction 150 mg, 12‐week maintenance 250 mg, 2‐week taper of 150 mg for 1 week and 50 mg for 1 week) or placebo for 15 weeks, and followed‐up for 4 weeks after end of treatment (Week 19). Additionally, all participants were offered a structured four‐session manual‐guided methamphetamine use CBT programme by trained and supervised therapists as standard care [20, 21].

### Study procedures

Participants were randomized in a 1:1 ratio between groups using variable block randomisation stratified by treatment site. A computer‐generated randomisation schedule was developed by an independent statistician and uploaded to the study database, with randomisation performed by site pharmacists. All other study staff were blinded to treatment allocation and to urine drug screen results.

Study visits occurred daily for the first 5 days, and then twice weekly for the remainder of the study. At each study visit, in‐person supervised medication was administered and in‐person urine samples were taken, vital signs and adverse events were recorded and unused medication returned and reconciled, together with medication adherence counselling [22]. Self‐reported methamphetamine use was recorded weekly. One urine per week was selected at random for analysis by immunoassay for amphetamine‐type substances (cut‐off of 300 ng/ml), followed by confirmation of drug type (methamphetamine, amphetamine or other) by gas chromatography–mass spectroscopy (cut‐off of 150 ng/ml) [23]. Long research visits occurred at baseline (Week 1), Weeks 5, 9 and 13 and at follow‐up 4 weeks following the end of medication (Week 19). Additional safety measures were administered at these visits to screen for psychosis (Brief Psychiatric Rating Scaling psychosis and hostility items [24], suicidality (Columbia Suicide Severity Rating Scale) [25] and cardiac adverse events [electrocardiogram (ECG)]. Participants were reimbursed with a supermarket voucher at screening and Weeks 1, 5, 9, 13 and 19. Reimbursements started at A$20 and increased by A$10 at each of the five subsequent visits (totalling a maximum per participant of A$270). Medical reviews were conducted at Weeks 5, 9 and 13, together with a post‐study treatment planning session prior to the end of the medication period (Week 15). Participants were withdrawn from study medication if a moderate or severe adverse event thought to be related to the study drug did not resolve after withholding the dose.

Modifications to the trial were necessary due to the COVID‐19 pandemic. The initial response to Australia's public health measures consisted of an HREC approved action plan (April 2020), reducing in‐person visits to once every 14 days with one supervised dose and up to 13 dispensed for self‐administration. Data collection by telephone was also permitted and up to three unsupervised urine samples collected at home. Face‐to‐face assessments were reduced to 1‐hour duration; ECGs were ceased. No trial data were used to inform these modifications, which were introduced to reduce the risk of infection by minimizing in‐person contact. Following the easing of COVID‐19 restrictions, a revised protocol (approved October 2020) was implemented. Data collection and supervised medication study visits reduced to two in the induction week and weekly thereafter. Sufficient study medication was dispensed at each visit for daily self‐administration until the next clinic appointment. Urine samples reduced to one per week for analysis.

An Independent Data Safety Monitoring Committee (IDSMC) monitored the conduct of the trial and supported the COVID‐19 modifications for the purpose of optimizing participant and staff safety.

### Measurements

#### Primary outcome

The primary efficacy measure was the between‐group difference in the number of days of self‐reported methamphetamine use in the past 28 days at the end of the 12‐week maintenance period (Weeks 9–13) using the time‐line follow back questionnaire (TLFB) [26].

#### Secondary outcomes

Secondary measures related to methamphetamine use were: total number of days of self‐reported methamphetamine use during the 12‐week treatment period (possible range = 0–84 days); mean longest period of abstinence during treatment period; percentage abstinent on self‐report for 21 days or greater; percentage of methamphetamine‐negative urine tests over 12 weeks (one a week, with missing tests considered positive) [27], and in the last 4 weeks of the 12‐week treatment period (of a total of four, with missing tests imputed as positive) [27]. The TLFB was administered at baseline (Day 1, Week 1, first dose), Week 5, Week 9, the primary end‐point of Week 13 and at follow‐up to Week 19, and self‐reported abstinence was confirmed by urine test.

#### Other measures

Other secondary measures administered at baseline and Weeks 5, 9, 13 (primary end‐point) and 19 (follow‐up) included: Visual Analog Scale for Craving [28], Amphetamine Withdrawal Questionnaire [29], Severity of Dependence Scale [30], TLFB for other substance use [26], Insomnia Severity Index [31], Drug Effects Questionnaire [32], Depression, Anxiety and Stress Scales‐21 (DASS‐21) [33], Patient Health Questionnaire‐15 (PHQ‐15) [34], World Health Organization's Quality of Life Brief version assessment (WHOQOL‐BREF) [35], treatment retention, the Opioid Treatment Index—injecting (OTI‐I) [36] and criminal behaviour (OTI‐C) [36] and Treatment Satisfaction Questionnaire for Medicines (TQSM) [37]. The Wender‐Utah Rating Scale (WURS) [38] to screen for childhood ADHD and the Enriched Social Support Index (ESSI) [39] as a measure of social support were administered at baseline, together with demographic data (Table 1).

**TABLE 1:** Participant characteristics.

	Placebo (*n* = 80)	Lisdexamfetamine (*n* = 75)	All (*n* = 155)
Age in years, mean (SD)	39.6 (9.44)	39.1 (9.21)	39.4 (9.30)
Current gender identity			
Male, *n* (%)	45 (56)	50 (67)	95 (61)
Female, *n* (%)	34 (43)	25 (33)	59 (38)
Different identity, *n* (%)	1 (1)	0 (0)	1 (1)
Aboriginal status			
Neither Aboriginal nor Torres Strait Islander, *n* (%)	71 (89)	67 (89)	138 (89)
Aboriginal and/or Torres Strait Islander, *n* (%)	8 (10)	7 (9)	15 (10)
Prefer not to say, *n* (%)	1 (1)	1 (1)	2 (1)
Current gender identity, sexual identity			
Male, straight or heterosexual, *n* (%)	29 (36)	34 (45)	63 (41)
Female, straight or heterosexual, *n* (%)	28 (35)	22 (29)	50 (32)
Male, gay or homosexual, *n* (%)	15 (19)	13 (17)	28 (18)
Female, lesbian or homosexual, *n* (%)	1 (1)	0 (0)	0 (0)
Male, other sexual identity, *n* (%)	2 (3)	3 (4)	5 (3)
Female, other sexual identity, *n* (%)	5 (6)	3 (4)	8 (5)
Other gender identity, other sexual identity, *n* (%)	1 (1)	0 (0)	1 (1)
Highest level of education			
Year 10 or below, *n* (%)	33 (41)	38 (51)	71 (46)
Year 12, *n* (%)	12 (15)	11 (15)	23 (15)
Trade/technical/vocational training, *n* (%)	18 (23)	14 (19)	32 (21)
University degree, *n* (%)	17 (21)	12 (16)	29 (19)
Income			
Full‐time work, *n* (%)	16 (20)	14 (19)	30 (19)
Part‐time/casual work, *n* (%)	10 (13)	9 (12)	19 (12)
Pension/benefit, *n* (%)	45 (38)	41 (55)	86 (55)
Other, *n* (%)	9 (11)	11 (15)	20 (13)
Housing			
Home owner, *n* (%)	8 (10)	19 (25)	27 (17)
Renting, state housing, *n* (%)	25 (31)	17 (23)	42 (27)
Renting, private landlord, *n* (%)	29 (36)	25 (33)	54 (35)
Other, *n* (%)	18 (23)	14 (19)	32 (21)
Social support (ESSI)			
Moderate/high, *n* (%)	59 (74)	57 (76)	116 (75)
Low, *n* (%)	21 (26)	18 (24)	39 (25)
Childhood ADHD WURS ≥ 46, *n* (%)	31 (39)	30 (40)	61 (39)
Days of methamphetamine use in last 28, mean (SD)	24 (4.6)	23 (5.6)	24 (5.1)
Age of first use (years), mean (SD)	23.6 (9.02)	22.1 (8.73)	22.9 (8.89)
Any injecting use, *n* (%)	34 (43)	42 (56)	76 (49)
Severity of Dependence Scale score, mean (SD)	10 (3.7)	9 (3.4)	10 (3.6)

Abbreviations: ADHD = attention deficit hyperactive disorder; ESSI = Enriched Social Support Index; SD = standard deviation; WURS = Wender‐Utah Rating Scale.

#### Adverse events

Adverse events were classified according to the preferred term and system organ class of the *Medical Dictionary for Regulatory Activities*, version 24.1 and evaluated for seriousness, severity and relatedness to study medication by site principal investigators, who were blinded to group allocation. Serious adverse events were evaluated for relatedness by the trial Chairperson (coordinating principal investigator), who was also blinded to group allocation. All adverse event data (and their group allocation) were reviewed by the IDSMC.

### Sample size

A sample size of 63 in each group was estimated to have 80% power at *P* < 0.05 to detect a between‐group mean difference of 4.5 days in past 28‐day methamphetamine use at week 13. Based on a previous Australian study [40] of participants in the existing New South Wales Stimulant Treatment Program (equivalent to the placebo control condition), we anticipated 9 days’ use out of the previous 28 with standard deviation (SD) of 9 days in the placebo group and 4.5 days of the previous 28, with SD of 9 days in the lisdexamfetamine group at the primary end‐point (week 13). Allowing for attrition of 30%, the recruitment target of 90 participants in each group was set.

### Statistical analysis

The full statistical analysis plan was finalised (18 July 2022) prior to unblinding the study and is provided in Supporting information, Data S1. In response to peer review, clinical site was included as a random‐effect component. All randomised participants who received at least one dose of study drug were analysed according to their randomized treatment allocation. All *P*‐values were from two‐sided tests and results were deemed statistically significant at *P* < 0.05. Analyses were performed using R version 4.1.2 (R Foundation for Statistical Computing) [41], with package glmmTMB [42] used to fit models, DHARMa [43] used to check model diagnostics and mice [44] used to perform and analyze multiple imputation.

#### Primary outcome

Analysis of the primary outcome was performed using a likelihood‐based mixed‐effects regression model with days of methamphetamine use at baseline, Weeks 5, 9, 13 and 19 used as the time‐varying dependent variable based on a beta‐binomial distribution for the number of days of methamphetamine use, a missing‐at‐random (MAR) assumption. The model fixed‐effects were (categorical) study visit, randomized treatment group and their interaction. Site‐specific random intercepts and treatment effects were included to allow for between‐site differences, such that the estimated overall treatment effect can be interpreted as that at the ‘average’ site. Participant‐specific random effects were included to account for the temporal correlation in the outcome measure. The mean difference between groups at baseline was fixed as zero using the constrained longitudinal data analysis (cLDA) model [45], with the parameter of primary interest being the difference between groups in the past 28‐day use at Week 13, adjusted for baseline use.

Model diagnostics using a dummy allocation list suggested a beta‐binomial model as the most suitable distribution for the primary outcome; full details are provided in Supporting information, Data S2. The difference between groups was calculated as the adjusted odds ratio (OR) and 95% confidence interval (CI) comparing the expected rates of methamphetamine use, and G‐computation [46] was used to convert this into an estimated mean difference in days of use.

Sensitivity analyses were conducted to assess the robustness of the results to different analysis approaches under a MAR assumption. First, alternatives to the primary analysis method were applied, while retaining the MAR assumption. Secondly, analyses were conducted assuming missing‐not‐at‐random (MNAR) with three imputation scenarios for missing data: ‘worst‐case’ assuming 28 days methamphetamine use, ‘jump‐to‐reference’ using data placebo arm data and return‐to‐baseline use. The results for the return‐to‐baseline MNAR assumption are included alongside the primary MAR analysis, with results for all sensitivity analyses reported in Supporting information, Data S2.

Pre‐specified analyses compared the treatment effect between subgroups of participants screening positive for childhood ADHD with the WURS [38], by level of social support (ESSI [39]) and by pandemic status.

Secondary analyses of the primary outcome used multiple imputation to target an on‐treatment estimand of the treatment effect, and a per‐protocol analysis to target a principal stratum estimand [47].

#### Secondary outcomes and other measures

Secondary outcomes and other measures were analysed by mixed‐effects regression models with participant‐specific random intercepts, site‐specific random intercepts and treatment effects and baseline differences fixed at zero where appropriate, with the response distribution chosen according to the type of measure.

#### Adverse events

Adverse events between groups were compared with Fisher's exact tests.

## RESULTS

### Study recruitment and retention

**FIGURE 1:**
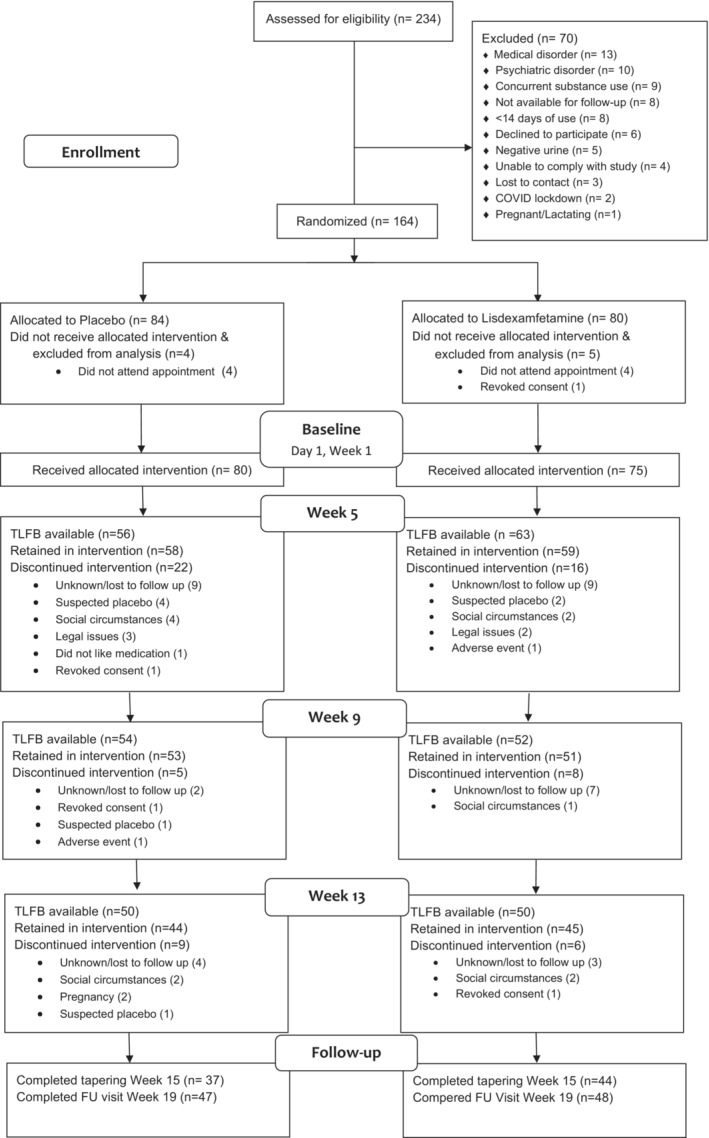
Consolidated Standards of Reporting Trials (CONSORT) diagram trial profile.

Participants were recruited from May 2018 to December 2021 from six specialist outpatient clinics in Adelaide, Melbourne, Newcastle and Sydney, Australia. Recruitment was suspended from March to October 2020 due to public health measures in response to the COVID‐19 pandemic requiring extension to the recruitment period. A study flow‐chart is presented as a CONSORT diagram in Figure 1. One hundred and sixty‐four were enrolled (62% male, 38% female, < 1% other; mean age 39 years; 84 in the placebo group and 80 in the lisdexamfetamine group); nine did not commence study medication post‐randomisation. A sample of 155 participants who received at least one dose of the study medication (80 in the placebo group and 75 in the lisdexamfetamine group) was retained for the primary analysis. Of these, a total of 100 participants (50 placebo, 50 lisdexamfetamine) completed the TLFB28 at the Week 13 time‐point, an attrition rate of 39.0%. Among these participants, the pooled standard deviation of the primary outcome was 9.48. Given that the target recruitment was not achieved, the attrition was higher than anticipated and the outcome was (slightly) more variable than anticipated, the power to detect a between‐group difference of 4.5 days of use in such a study is 65.2%, lower than planned.

Participants [95 male (61%), 59 female (38%), one other (1%); mean age = 39.6 years (SD = 9.30)] used methamphetamine a mean of 23.7 days (SD = 5.13) of the previous 28 at baseline (Table 1). Thirty‐nine per cent (*n* = 61) of participants scored positive (≥ 46) for childhood symptoms of ADHD using the WURS [38]. Both groups had a similar estimated average attendance in CBT: 1.0 sessions (95% CI = 0.7–1.4) in the placebo and 1.2 sessions (95% CI = 0.9–1.5) in the lisdexamfetamine groups, respectively (OR = 1.0, 95 CI = 0.4–2.2; *P =* 0.92).

One‐hundred and eighteen participants were enrolled pre‐pandemic [97 (63%) reached the primary endpoint prior to pandemic measures and 21 (14%) were on study at the onset], and 37 (24%) participants were enrolled after post‐pandemic restrictions were eased. Compared to those randomised prior to pandemic onset, the post‐pandemic cohort showed an increase in the proportion of university educated (14–35%; *P =* 0.002) and gay men (13–23%; *P =* 0.04), with one inner‐city site (site 4) representing a larger proportion of overall recruitment during this time (18–32%; *P =* 0.02).

**FIGURE 2:**
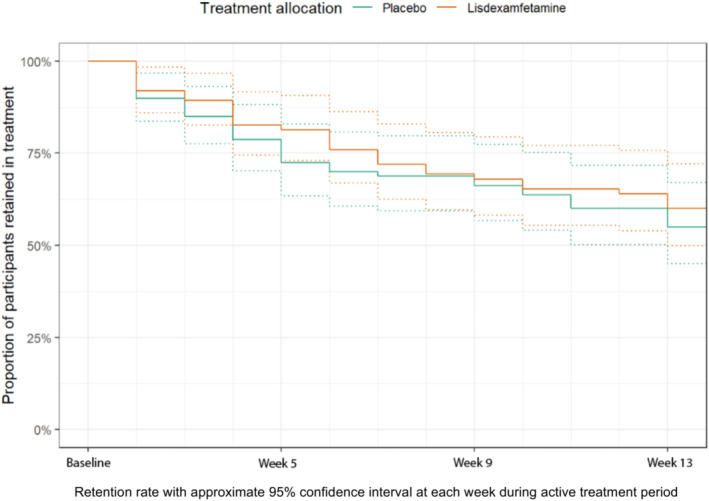
Retention rate.

At the primary end‐point (Week 13), the primary outcome measure was collected for 50 (60%) in the placebo group and 50 (63%) in the lisdexamfetamine group. Eighty‐nine participants (57%) were retained in treatment at Week 13, with no difference between groups: 55% (95% CI = 45–67%) in the placebo group compared to 60% (95% CI = 50–72%) in the lisdexamfetamine group (*P =* 0.64) (Figure 2). There were no significant differences in treatment retention between those enrolled pre‐ and post‐pandemic onset.

### Primary outcome

**FIGURE 3:**
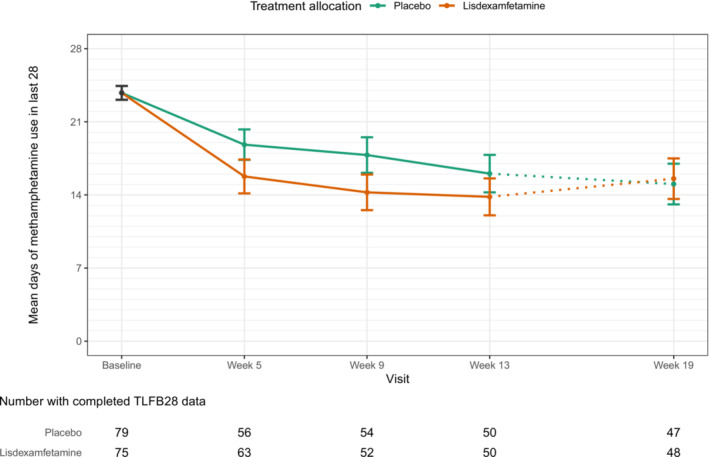
Primary outcome (days of methamphetamine use).

An OR of 0.72 (95% CI = 0.29–1.82; *P* = 0.49) was estimated for the difference between the lisdexamfetamine and placebo groups in the expected daily probability of methamphetamine use in the 28 days prior to the week 13 visit (Figure 3), the primary outcome, corresponding to an estimated difference of 2.2 fewer days use in the lisdexamfetamine group (95% CI = –0.5 to 5.0, Table 2), was a weak finding. These results, which employ a MAR assumption, are consistent with sensitivity analyses under a ‘return‐to‐baseline’ MNAR assumption, where the estimated OR was 0.68 (95% CI = 0.36–1.27; *P* = 0.23) and difference in days of use was 2.2 (95% CI = –0.1 to‐‐ 4.4).

**TABLE 2:** Methamphetamine use outcomes.

	Baseline	Baseline‐ and site‐adjusted estimates				
	Pooled mean (CI)	Placebo mean (CI)	Lisdexamfetamine mean (CI)	Difference (CI)	OR/means ratio (MR) (CI)	*P*‐value
Primary outcome						
Primary analysis (MAR assumption) Days of use of previous 28 days at Week 13 (CI)	23.8 (23.1, 24.4)	16.0 (14.2, 17.8)	13.8 (12.0, 15.6)	−2.2 (−5.0, 0.5)	OR 0.72 (0.29, 1.82)	0.489
Sensitivity analysis (return‐to‐baseline MNAR assumption) Days of use of previous 28 days at Week 13 (CI)	23.6 (22.9, 24.2)	19.3 (18.0, 20.7)	17.2 (15.8, 18.6)	−2.2 (−4.4, 0.1)	OR 0.68 (0.36, 1.27)	0.225
Secondary outcomes						
Days of use over 12 week treatment period (CI)^a^	NA	52.7 (49.1, 56.3)	43.8 (40.1, 47.6)	−8.8 (−15.0, −2.7)	NA	0.005
Longest period of abstinence, mean days (CI)	NA	8.3 (6.7, 10.0)	10.9 (9.2, 12.6)	2.6 (−0.5, 5.7)	MR 1.31 (0.89, 1.92)	0.169
Percentage achieving 21 days of abstinence (CI)	NA	4.5% (0.0%, 9.0%)	12.8% (4.9%, 20.7%)	NA	OR 6.16 (0.33, 114.05)	0.220
Proportion of negative urines, 12 weeks (CI)	NA	3.6% (1.4%, 5.8%)	3.6% (1.4%, 5.9%)	0.1% (−2.8%, 2.9%)	OR 1.02 (0.41, 2.54)	0.962
Proportion of negative urines, last 4 weeks (CI)	NA	4.5% (0.9%, 8.1%)	3.2% (0.2%, 6.3%)	−1.3% (−5.7%, 3.2%)	OR 0.69 (0.11, 4.32)	0.688
Participants retained at Week 5 (CI)^b^		72% (63%, 83%)	81% (73%, 91%)	NA	NA	0.266
Participants retained at Week 9 (CI)^b^		66% (57%, 77%)	68% (58%, 79%)	NA	NA	0.952
Participants retained at Week 13 (CI)^b^		55% (45%, 67%)	60% (50%, 72%)	NA	NA	0.641
Craving Visual Analogue Scale score (CI)	65.2 (61.6, 68.9)	48.1 (40.4, 55.8)	41.1 (33.4, 48.8)	−7.0 (−18.3, 4.4)	OR 0.75 (0.33, 1.75)	0.510
Amphetamine Withdrawal Scale score (CI)	17.0 (16.2, 17.9)	14.6 (12.8, 16.4)	12.1 (10.4, 13.8)	−2.5 (−5.1, 0.1)	OR 0.78 (0.50, 1.22)	0.275
Severity of Dependence Scale score (CI)	9.7 (9.3, 10.1)	7.2 (6.3, 8.1)	6.6 (5.7, 7.5)	−0.5 (−1.9, 0.8)	OR 0.85 (0.49, 1.48)	0.561
Days of injecting in previous 28 (CI)	NA	4.7 (3.5, 6.0)	5.0 (3.9, 6.1)	0.3 (−1.8, 2.4)	OR 1.19 (0.31, 4.52)	0.798

*Note*: Estimated means in each treatment arm and the difference between arms (with 95% CIs) were derived using G‐computation from repeated measures regression models adjusted for site and with each arm constrained to have equal means at baseline. The primary effect measure [odds ratio (OR) or mean ratio (MR)] and its corresponding *P*‐value are provided.

Abbreviations: CI = 95% confidence interval; MAR = missing‐at‐random; MNAR = missing‐not‐at‐random; NA = not applicable.

^a^ Derived from primary model that estimates separate odds ratios at weeks 5, 9 and 13: there is no common OR estimate.

^b^ Analysed with χ^2^ test, no between‐group difference calculated.

### Secondary outcomes

#### Methamphetamine use outcomes

There was a difference of 8.8 fewer days between the estimated average number of days of methamphetamine use during the 84 days of the 12‐week treatment period in the lisdexamfetamine group compared to the placebo group, as shown in Table 2. This difference was not sustained on follow‐up 4 weeks after study medication cessation (week 19, Figure 3). There were no significant between‐group differences for the proportion of participants with continuous 21‐day abstinence or for mean longest period of abstinence, proportion of methamphetamine‐negative urines, or measures of craving, withdrawal or severity of dependence (Table 2).

Sensitivity analyses under both MAR and MNAR assumptions were broadly consistent with the primary results (see Supporting information, Data S2). In a principal stratum estimand (per‐protocol) analysis of the 89 participants who completed the 12‐week treatment regimen, between‐group differences were not significant at the primary end‐point of week 13 (OR = 0.70, 95% CI = 0.22–2.23; *P =* 0.54). Similarly, the on‐treatment estimand (the treatment effect if full adherence to the 12‐week treatment regimen was achieved for all 155 participants) was not significant at week 13 (OR = 0.72, 95% CI = 0.32–1.60; *P =* 0.42). Pre‐planned subgroup analyses showed no significant differences in the estimated treatment effect between participant groups defined by their pandemic status, childhood ADHD status or level of social support.

#### Treatment satisfaction

The TSQM showed significantly higher scores for effectiveness and global satisfaction for the lisdexamfetamine group and no difference on measures for side‐effects or convenience (Table 4).

#### General health and quality of life

General health and psychosocial functioning outcomes are shown in (Table 4). There was weak evidence of greater end‐of‐treatment overall health‐related quality of life in the lisdexamfetamine group.

#### Adherence

There were no significant between‐group differences in adherence to supervised doses (61% of the placebo group and 67% of the lisdexamfetamine group, OR= 1.32, 95% CI = 0.86–2.02; *P* = 0.20) or in adherence among retained participants prior to or after pandemic onset.

#### Extra‐medical use liability

The Drug Effects Questionnaire elicited higher drug‐liking scores in the lisdexamfetamine group [32 mm on a 100‐mm visual analogue scale (VAS), interquartile range (IQR) 9.5–62.5 mm] compared to placebo (30 mm, IQR = 0–50.8 mm; OR = 3.78, 95% CI = 1.31–10.87; *P* = 0.028), as well as on questions on similarity with methamphetamine (15 mm, IQR = 6.15–51.5 versus 0 mm, IQR = 0–36.5 mm; OR = 9.30, 95% CI = 2.75–31.50; *P* < 0.001) and the price participants would pay for the medication (AU$20 IQR AU$0–AU$25, placebo group AU$0 IQR = AU$0–AU$10, OR = 15.62, 95% CI = 1.89–129.08; *P* = 0.011).

#### Other drug use

There were no between‐group differences in other drug use on self‐report or urine drug screen.

#### Testing the study blind

There was a statistically significant greater proportion of correct guesses (*P* < 0.001) for treatment allocation among retained participants (143 placebo group, 155 lisdexamfetamine group), with 80% (*n* = 122) of guesses in the lisdexamfetamine group being correct, compared to 62% (*n* = 88) in the placebo group.

### Adverse events

There were eight serious adverse events: five in the lisdexamfetamine group and three in the placebo group (Table 3). Adverse events were mostly mild–moderate. Nausea was the only adverse event that occurred more frequently (*P* < 0.05) among participants in the lisdexamfetamine group than in the placebo group (16 versus 5%; *P* = 0.03). A *post‐hoc* analysis of cardiovascular data showed no strong evidence of between‐group differences in mean heart rate or systolic blood pressure at any time‐point during the active treatment period, while mean diastolic blood pressure was significantly higher in the lisdexamfetamine group at Weeks 5 and 8 (maximum difference at Week 8 of 4.0 mmHg, 95% CI = 1.0–7.0; *P* = 0.009).

**TABLE 3:** Adverse events.^a^

	Placebo (*n* = 80)	Lisdexamfetamine (*n* = 75)	*P*
Total number of serious adverse events^b^	3	5	
Participants with at least one serious adverse event^c^	3 (4%)	4 (5%)	0.71
Participants with any adverse event, *n* (%)	58 (73%)	56 (75%)	0.86
Participants with adverse events by system organ class and preferred term, *n* (%)^d^			
Cardiac disorders			
Palpitations	2 (3%)	4 (5%)	0.43
Tachycardia	4 (5%)	4 (5%)	1
Gastrointestinal disorders			
Abdominal discomfort	0	2 (3%)	0.24
Abdominal pain upper	1 (1%)	2 (3%)	0.61
Diarrhoea	4 (5%)	4 (5%)	1
Dry mouth	3 (4%)	3 (4%)	1
Gastro‐oesophageal reflux disease	5 (6%)	3 (4%)	0.72
Nausea	4 (5%)	12 (16%)	0.03
Tongue discomfort	3 (4%)	4 (5%)	0.71
Toothache	3 (4%)	4 (5%)	0.71
Vomiting	1 (1%)	4 (5%)	0.20
General disorders and administration site conditions			
Fatigue	7 (9%)	4 (5%)	0.54
Infections and infestations			
Nasopharyngitis	2 (3%)	4 (5%)	0.43
Injury, poisoning and procedural complications			
Overdose	0	2 (3%)	0.24
Musculoskeletal and connective tissue disorders			
Arthralgia	1 (1%)	2 (3%)	0.61
Back pain	6 (8%)	3 (4%)	0.50
Nervous system disorders			
Dizziness	2 (3%)	4 (5%)	0.43
Headache	21 (26%)	26 (35%)	0.30
Paraesthesia	1 (1%)	2 (3%)	0.61
Psychiatric disorders			
Agitation	3 (4%)	2 (3%)	1
Anger	1 (1%)	2 (3%)	0.61
Anxiety	4 (5%)	5 (7%)	0.74
Depressed mood	2 (3%)	2 (3%)	1
Depression	2 (3%)	2 (3%)	1
Emotional distress	5 (6%)	3 (4%)	0.72
Insomnia	3 (4%)	6 (8%)	0.32
Panic attack	2 (3%)	3 (4%)	0.67
Suicidal ideation	3 (4%)	2 (3%)	1
Respiratory, thoracic and mediastinal disorders			
Cough	2 (3%)	4 (5%)	0.43
Oropharangeal pain	5 (6%)	4 (5%)	1
Rhinorrhea	2 (3%)	2 (3%)	1
Skin and subcutaneous tissue disorders			
Hyperhydrosis	0	2 (3%)	0.23
Rash	4 (5%)	2 (3%)	0.68
Vascular disorders			
Hot flush	1 (1%)	2 (3%)	0.61
Hypertension (SBP > 160 and/or DBP > 100 mmHg)	4 (5%)	3 (4%)	1

Abbreviations: DBP = diastolic blood pressure; SBP = systolic blood pressure.

^a^ Adverse events were classified according to the preferred term and system organ class of the *Medical Dictionary for Regulatory Activities*, version 24.1.

^b^ The serious adverse events were: facial cellulitis, intentional overdose, overdose ×2, bipolar I disorder in the lisdexamfetamine group; schizophrenia, substance‐induced psychosis and Crohn's disease in the placebo group. Seven serious adverse events were reported as serious because they resulted in inpatient hospitalization or prolongation of hospitalization; one overdose was reported as serious because it was life‐threatening.

^c^ One participant in the lisdexamfetamine group experienced two serious adverse events: facial cellulitis and bipolar I disorder.

^d^ Adverse events reported here are those that occurred in 3% of more of participants in the lisdexamfetamine group or for which *P* < 0.05 on pairwise comparison.

**TABLE 4:** Other outcomes.

	Baseline	Week 13 (baseline‐ and site‐adjusted)				
	Pooled mean (CI)	Placebo Mean (CI)	Lisdexamfetamine Mean (CI)	Difference (CI)	Odds ratio (CI)	*P*‐value
TSQM^a^						
Effectiveness score	NA	37.1 (29.9, 44.3)	59.4 (52.7, 66.1)	22.3 (11.3, 33.2)	2.89 (1.67, 5.02)	< 0.001
Side effects score	NA	97.2 (95.2, 99.3)	95.8 (93.2, 98.4)	−1.5 (−5.3, 2.4)	0.54 (0.11, 2.68)	0.453
Convenience score	NA	76.2 (71.1, 81.4)	73.9 (68.6, 79.2)	−2.3 (−10.4, 5.7)	0.92 (0.58, 1.47)	0.737
Global satisfaction score	NA	47.0 (38.4, 55.6)	72.0 (65.5, 78.5)	25.0 (13.0, 37.0)	3.80 (1.93, 7.47)	< 0.001
Insomnia Severity Index score	11.6 (10.9, 12.2)	8.8 (7.5, 10.0)	9.1 (7.9, 10.3)	0.3 (−1.5, 2.1)	1.04 (0.73, 1.48)	0.844
PHQ‐15 score	7.6 (7.1, 8.1)	6.3 (5.3, 7.3)	5.6 (4.7, 6.6)	−0.6 (−2.1, 0.8)	0.84 (0.59, 1.18)	0.311
DASS‐21						
Depression score (normal ≤9)	14.6 (13.6, 15.5)	12.5 (10.5, 14.4)	9.8 (8.0, 11.5)	−2.7 (−5.5, 0.1)	0.71 (0.43, 1.19)	0.195
Anxiety score (normal ≤7)	11.1 (10.4, 11.8)	8.7 (7.3, 10.1)	7.1 (5.7, 8.5)	−1.6 (−3.7, 0.5)	0.76 (0.51, 1.13)	0.178
Stress score (normal ≤14)	16.4 (15.5, 17.4)	13.7 (11.6, 15.7)	11.1 (9.3, 13.0)	−2.6 (−5.5, 0.4)	0.72 (0.49, 1.06)	0.097
WHOQOL‐BREF						
Overall Quality of Life score^b^	NA	NA	NA	NA	1.31 (0.40, 4.25)	0.652
Overall health score^b^	NA	NA	NA	NA	3.09 (0.98, 9.77)	0.054
Physical health score	58.0 (56.4, 59.7)	60.5 (56.9, 64.0)	65.3 (61.9, 68.8)	4.9 (−0.4, 10.1)	1.24 (0.84, 1.82)	0.278
Psychological score	46.6 (45.0, 48.2)	50.6 (47.1, 54.1)	55.4 (52.0, 58.8)	4.8 (−0.4, 10.0)	1.23 (0.96, 1.58)	0.109
Social relationships score	45.0 (42.6, 47.3)	48.1 (42.9, 53.3)	52.1 (47.2, 57.1)	4.0 (−3.7, 11.7)	1.25 (0.81, 1.92)	0.307
Environment score	58.7 (57.0, 60.4)	58.4 (54.8, 62.1)	65.1 (61.5, 68.7)	6.7 (1.2, 12.1)	1.36 (0.99, 1.88)	0.060
OTI‐crime score	1.1 (0.9, 1.2)	0.6 (0.4, 0.9)	0.4 (0.2, 0.7)	−0.2 (−0.6, 0.2)	0.72 (0.33, 1.55)	0.401
OTI‐injecting drug use score	2.8 (2.5, 3.2)	1.5 (0.9, 2.1)	1.3 (0.8, 1.7)	−0.3 (−1.0, 0.5)	0.97 (0.31, 3.05)	0.961

Abbreviations: DASS = Depression, Anxiety, and Stress Scales; OTI = Opioid Treatment Index; PHQ = Patient Health Questionnaire; TSQM = Treatment Satisfaction Questionnaire for Medication; WHOQOL‐BREF = World Health Organization's Quality of Life Assessment Brief Version.

^a^ TSQM was not administered at baseline. 36 participants (23 placebo, 13 lisdexamfetamine) who did not complete any post‐baseline assessments are excluded.

^b^ Five‐level ordinal scales, analyzed using a mixed‐effects cumulative logistic regression model. Odds ratio presented for odds of a higher response, lisdexamfetamine versus placebo.

## DISCUSSION

This study reports the first outpatient randomised clinical trial for lisdexamfetamine for the treatment of methamphetamine dependence. There was a reduction in days of methamphetamine use among those receiving daily 250 mg lisdexamfetamine compared with placebo during the 12‐week treatment period, but not at the primary end‐point of past‐28 day use at Week 13. Lisdexamfetamine treatment reduced frequency of use early in treatment, but the benefit was not sustained for the duration of the study. Exploring the characteristics of early responders will be useful to understand who may benefit from agonist therapies. Importantly, there were no unexpected safety concerns at this dose, and participants receiving lisdexamfetamine reported significantly higher ratings of treatment effectiveness and satisfaction. Reduced use is consistent with a recent meta‐analysis of randomised controlled trials (RCTs) which showed that, after removing studies with high risk of bias, prescription psychostimulants (particularly high dose such as methylphenidate > 162 mg/day) reduce amphetamine‐type stimulant use [8]. Experienced specialist clinicians could consider off‐label prescription of lisdexamfetamine 250 mg with close monitoring of risks and benefits, in line with current guidelines for prescription psychostimulants for methamphetamine use disorder [48].

There were a number of limitations to the study. First, 57% (*n* = 89) of the study sample remained on study medication to the primary end‐point; 50 participants in each group (60% placebo and 63% lisdexamfetamine) had complete data at primary end‐point in each group. This level of retention is consistent with other outpatient studies of 12 weeks of stimulant medications for methamphetamine use disorder [5], and exceeds typical retention in usual community care. Nonetheless, the study was underpowered (re‐calculated at 65% to detect a difference of 4.5 days), and although the study gained some power through its analysis method, specifically that partial information from randomised participants with missing outcomes at Week 13 (with adjustment for site as a prognostic factor for methamphetamine use) was used. Higher rates of retention would improve power and reduce statistical uncertainty. As contingency management, a treatment in itself, reduces treatment dropout rate [3], future studies could test the effectiveness of combination contingency management and pharmacotherapy.

Secondly, the study was impacted by the COVID‐19 pandemic. Extended public health measures resulted in recruitment suspension of 9 months and the loss of a study site. A subsequent protocol change increased the use of telephone follow‐up and reduced the frequency of contact and medication supervision. Australian federal and state border closures and restrictions on movement in the community resulted in reduced availability of methamphetamine [49], which may have contributed to changes in participant behaviour. We did not detect a difference in primary outcome between those enrolled pre‐ and post‐pandemic onset, although numbers were small, with 37 (24%) enrolled post‐pandemic.

Other limitations to the study include measures of adherence to the medication which relied mainly upon self‐report.^:^ Although the study duration of 12 weeks is commonly accepted for dependence treatment trials [5], the trial was not designed to test optimal duration of therapy nor tapering regimens, and future research is required to consider these elements. Lisdexamfetamine is used in the treatment of ADHD and a large proportion (39%) of the participant population screened positive for childhood ADHD. As the study was not powered to detect a differential effect of the drug for this group, future research could consider stratifying by ADHD status [50]. Although the study was proposed by a person with living experience of methamphetamine dependence, the study design, implementation and analysis had limited consumer engagement increasingly recognized as important for research quality and relevance [51]. The fixed dose design was informed by our group's pilot escalation study [14]; nonetheless, it did not allow for individualization of dose, so we can draw no conclusions regarding safety and efficacy of higher doses. Finally, blinding for psychoactive medications is challenging, and given the proportion of participants who correctly guessed their allocation, participant expectancy may also affect these results.

There were five serious adverse events in the lisdexamfetamine group. Most adverse events were mild or moderate; nausea was more frequent in the lisdexamfetamine group. *Post‐hoc* analysis detected transient mild (< 4 mmHg) increases in diastolic blood pressure at two time‐points. Future investigation of lisdexamfetamine 250 mg daily requires continued cautious cardiovascular and neuropsychiatric monitoring, given the risks of adverse outcomes associated with methamphetamine use [52] and the known adverse event profile of prescribed psychostimulants.

The proportion of urinalyses positive for methamphetamine is the most commonly reported outcome measure in RCTs of psychostimulants for amphetamine type stimulant use disorder [9]. We selected a primary outcome of difference in days of use. Self‐report is a sufficiently valid measure [53], and the combination of urine toxicology and self‐report enhances the validity of self‐reported substance use [54]. The study design precluded verification of self‐reported use days by weekly urinalysis results due to differences in the periodicity in the two data sources. Reduced frequency of use is a valid outcome measure associated with improved drug use, health and wellbeing [55]. Nonetheless, there is no consensus on clinically meaningful reduction in use [56]. There is, however, growing recognition of participant‐reported outcome measures as alternative end‐points [57, 58]. We found positive findings regarding self‐reported treatment effectiveness and drug effect. This aspect of the treatment might be important for treatment uptake and underscores an imperative for stronger efficacy data.

Lisdexamfetamine 250 mg daily, relative to placebo, is associated with reduction of use over a 12‐week treatment period but not the last 4 weeks of treatment among people with methamphetamine dependence using on at least 14 of the previous 28 days. Lisdexamfetamine was associated with participant‐reported treatment effectiveness and overall satisfaction. The place of lisdexamfetamine in the treatment of methamphetamine dependence remains inconclusive.

## AUTHOR CONTRIBUTIONS

**Nadine Ezard:** Conceptualization (equal); formal analysis (lead); funding acquisition (supporting); investigation (equal); project administration (supporting); writing—original draft (supporting). **Brendan Clifford:** Data curation (equal); formal analysis (supporting); funding acquisition (supporting); project administration (supporting); writing—original draft (lead). **Krista J. Siefried:** Formal analysis (supporting). **Robert Ali:** Conceptualization (supporting); funding acquisition (supporting). **Adrian Dunlop:** Conceptualization (supporting); funding acquisition (supporting); investigation (lead). **Rebecca McKetin:** Conceptualization (supporting); formal analysis (supporting); funding acquisition (supporting). **Raimondo Bruno:** Conceptualization (supporting); funding acquisition (supporting). **Andrew Carr:** Conceptualization (supporting); funding acquisition (supporting). **James Ward:** Conceptualization (supporting); funding acquisition (supporting). **Michael Farrell:** Conceptualization (supporting); funding acquisition (supporting). **Robert Graham:** Funding acquisition (supporting); investigation (equal). **Paul Haber:** Funding acquisition (supporting); investigation (equal). **Dan Lubman:** Funding acquisition (supporting); investigation (equal). **Mark W. Donoghoe:** Formal analysis (lead). **Nick Olsen:** Formal analysis (supporting). **Amanda Baker:** Conceptualization (supporting). **Michelle Hall:** Data curation (equal); project administration (lead). **Shalini Arunogiri:** Investigation (equal). **Nicholas Lintzeris:** Conceptualization (equal); funding acquisition (lead).

## DECLARATION OF INTERESTS

D.L. is supported by a NHMRC Investigator Grant (1196892). M.F. has received unrestricted funding for research purposes from Indivior and Sequiiris. M.M. has been an advisory board member for AbbVie Australia and Pfizer Australia, both unrelated to this project. N.L. has received funding from NHMRC, Camurus AB and Indivior for unrelated research. R.B. was an investigator on an untied education grant from Mundipharma to conduct post‐marketing surveillance on oxycodone and an untied educational grant from Reckitt‐Benckiser to develop a scale to identify extra medical use of pharmaceutical opioids. S.A. has received speaker honoraria from Camurus, Indivior, Gilead and Janssen for work unrelated to this study. All other authors have no relevant conflicts to declare.

## CLINICAL TRIAL REGISTRATION

Australia New Zealand Clinical Trial registry: ACTRN12617000657325 prospectively registered May 8, 2017.

## Supporting information


**Data S1.** Supporting information.

**Data S2.** Supporting information.

## Data Availability

Data supporting the findings of this study are available upon reasonable request from the corresponding author.
