# Supplementary material for: Lisdexamfetamine in the treatment of methamphetamine dependence: A randomised, placebo‐controlled trial
Source: Addiction. 2024 Dec 19;120(7):1345–59. doi: 10.1111/add.16730 (PMC12128569; doi:10.1111/add.16730)
Supplement: Supplementary file 1 — Data S1. Supporting information. [file ADD-120-1345-s002.pdf]

Statistical Analysis Plan

The LIMA Study

NHMRC Application ID: APP1109466

Universal Trial Number (UTN): U1111-1195-2142

Australian New Zealand Clinical Trials Registry: ACTRN12617000657325

VERSION 1.0 WITH AMENDMENT 1; 1 SEPTEMBER 2022

Document history

Trial Full Title	A randomised double blind placebo controlled study of lisdexamfetamine for the treatment of methamphetamine dependence (LIMA trial)
Trial Chief Investigator	Nadine Ezard
Protocol version	V8.0 (Amendment 7) 16Aug2020
SAP version	V1.0 with Amendment 1
SAP version date	18 July 2022 (Amendment 1: 1 September 2022)
SAP authors	Zhixin Liu (statistician), Mark Donoghoe (statistician)

Approvals

Name	Role on study	Signature	Date
Prof Nadine Ezard	Principal investigator		8 September 2022
Dr Mark Donoghoe	Statistician		8 September 2022

Amendments

Amendment	Date	Summary of changes	Reason
1	1 September 2022<br><br>*This amendment was approved after unblinding but before conducting the affected subgroup analyses.*	Section 4.3.3.3:<br>".. participants' "pandemic status" is classified into three categories based on the timing of randomisation ~~and completion or withdrawal~~: (1) reached primary endpoint at week 13 ~~or dropped out~~ before pandemic onset; (2) randomised and ~~on study~~ not yet reached primary endpoint at pandemic onset; (3) randomised after pandemic onset."	Avoid using post-randomisation information (date of withdrawal) to define subgroups
		Section 4.3.3.5:<br>Childhood ADHD status (baseline WURS score ≥ ~~36~~46)	Correct typo

Table of contents

1. INTRODUCTION 7
1.1. Background 7
1.2. Study synopsis 7
2. STUDY OBJECTIVES 8
3. OVERALL STUDY DESIGN 9
3.1. Study population 9
3.1.1. Inclusion criteria 9
3.1.2. Exclusion criteria 9
3.2. Intervention 10
3.3. Randomisation 10
3.4. Blinding 10
3.5. Study endpoints 11
3.5.1. Primary endpoint 11
3.5.2. Secondary endpoints 11
3.5.3. Safety endpoint 12
3.6. Sample size 12
3.7. Independent Data Safety Monitoring Committee (IDSMC) 13
3.8. Data collection and security 13
4. STATISTICAL METHODS 14
4.1. General considerations 14
4.1.1. Analysis principles 14
4.1.2. Analysis dataset 14
4.1.3. Missing data handling 15
4.2. Summary of study data 15
4.2.1. Protocol deviations 15
4.2.2. Participant disposition 15
4.2.3. Participant characteristics 15
4.2.4. Concomitant therapy 16
4.2.5. Treatment adherence 16
4.3. Analysis of primary outcome 16

4.3.1. Analysis method ... 16
4.3.2. Supplementing missing TLFB28 data with available TLFB7 data ... 16
4.3.3. Supportive and sensitivity analyses ... 17
4.3.3.1. Sensitivity to missing data assumptions ... 17
4.3.3.2. Sensitivity to baseline methamphetamine use cut-off ... 19
4.3.3.3. COVID-19 pandemic related modifications ... 19
4.3.3.4. Supplementary analysis ... 19
4.3.3.5. Subgroup analyses ... 20
4.4. Analysis of secondary outcomes ... 20
4.5. Analysis of safety outcomes ... 22
5. REFERENCES ... 23

List of abbreviations

**Abbreviation**	**Definition**
AE	Adverse Event
AWQ	Amphetamine Withdrawal Questionnaire
BPRS	Brief Psychiatric Rating Scale
CBT	Cognitive Behavioural Therapy
DASS	Depression & Anxiety Survey Scale
DEQ-5	Drug Evaluation Questionnaire
ESSI	ENRICHD Social Support Inventor
ISI	Insomnia Severity Index
ITT	Intention-to-treat
LDX	Lisdexamfetamine
MAR	Missing at random
MMRM	Mixed model for repeated measures
MNAR	Missing not-at-random
MoCA	MoCA, Montreal Cognitive Assessment
OTI-C	Opiate Treatment Index – Criminality section
OTI-I	Opiate Treatment Index - Injecting drug use section
PHQ-15	Patient Health Questionnaire-15
PPS	Per protocol set
RAVLT	Rey Auditory Verbal Learning Task
RCG	Readiness to Change Questionnaire

RVIP	Rapid Visual Information Processing
SAE	Serious Adverse Event
SAP	Statistical Analysis Plan
SDS	Severity of Dependence Scale
SF-12	Short Form-12 Health Survey
TEAE	Treatment Emergent Adverse Events
TSQM	Treatment Satisfaction Questionnaire for Medication
UDS	Urine Drug Screen
VAS	Visual Analogue Scale
WTAR	Wechsler Test of Adult Reading.
WURS	Wender-Utah Rating Scale

1. INTRODUCTION

1.1. BACKGROUND

Methamphetamine dependence is a growing public health concern. There is currently no pharmacotherapy approved for methamphetamine dependence. Lisdexamfetamine (LDX) dimesylate, used in the treatment of attention-deficit hyperactivity disorder and binge eating disorder, has potential as an agonist therapy for methamphetamine dependence, and possible benefits of reduced risk of aberrant use due to its novel formulation. We hypothesise that oral LDX treatment will result in significantly reduced methamphetamine use compared to placebo in people who are dependent on methamphetamine.

1.2. STUDY SYNOPSIS

The LIMA trial [1] is a multi-center double blinded randomised controlled trial designed to evaluate the efficacy of 12 weeks of daily LDX 250mg in reducing methamphetamine use. The target sample is 180 adults with methamphetamine dependence of ≥12 months who report use of ≥14 days out of the previous 28. Eligible participants were randomised in a 1:1 ratio to a 15-week intervention consisting of either induction (1 week of 150 mg LDX), maintenance (12 week of 250mg LDX) and reduction (1 week of 50mg LDX, 1 week of 50mg LDX); or placebo. All participants were given access to four sessions of cognitive–behavioural therapy as treatment as usual and received a follow up visit 4 weeks post treatment (week 19).

2. STUDY OBJECTIVES

LIMA is a randomised control trial designed with the primary objective to examine the efficacy of oral LDX in reducing methamphetamine use over a 12-week maintenance course of 250mg LDX daily comparing to placebo, and to examine the safety of oral LDX in people who are dependent on methamphetamine.

Secondary objectives are:

- To examine changes in physical and mental health, cognitive and psychosocial functioning and well-being in the study population between those taking oral LDX compared to placebo.
- To examine differences in retention rates in the study population between those taking oral LDX compared to placebo.
- To examine differences in amphetamine cravings, withdrawal and severity of dependence in the study population between those taking oral LDX compared to placebo.
- To examine differences in use of alcohol and other drugs in the study populations between those taking oral LDX compared to placebo.
- To examine differences in bloodborne virus transmission risk behaviour in the study population between those taking oral LDX compared to placebo.
- To examine difference in criminality in the study population between those taking oral LDX compared to placebo.
- To examine the abuse liability profile of oral LDX in people who are dependent on methamphetamine.

3. OVERALL STUDY DESIGN

This study is a multicentre, double-blind, randomised placebo controlled, parallel design trial, comparing a 12-week maintenance course of 250mg LDX daily to placebo in the targeted 180 participants. Eligible participants were randomised in a 1:1 ratio to an intervention or placebo group.

3.1. STUDY POPULATION

The study population is treatment-seeking adults with long-standing (≥12 months) methamphetamine dependence.

3.1.1. Inclusion criteria

1. Provide written, informed consent to participate in the study.
2. Aged 18 to 65 years.
3. Be treatment-seeking for methamphetamine use.
4. Meet ICD-10 criteria for methamphetamine dependence for at least twelve months.
5. Self-report methamphetamine use of ≥14 days out of the previous 28.
6. Have one urine drug screen (UDS) positive for methamphetamine at screening.
7. Be willing and able to comply with the requirements of the study.
8. The ability to store study medication securely.

3.1.2. Exclusion criteria

1. Current effective counselling-based treatment for methamphetamine dependence.
2. Current pharmacotherapy treatment for opioid dependence.
3. Use of prescription stimulant medication (e.g., dexamphetamine, modafinil, methylphenidate) in the previous four weeks (discuss with Trial Chairperson before excluding).
4. Current dependent use of alcohol or non-prescribed substances other than amphetamines, diagnosed by specialist clinical assessment against ICD-10 criteria, which in the opinion of the investigator would interfere with participation in the study.
5. Currently lactating or pregnant, or of childbearing potential and not willing to avoid becoming pregnant during the study.
6. Sensitivity or previous adverse reaction to LDX.
7. Current, severe medical disorder (e.g., cardiovascular disease, uncontrolled hypertension,

peripheral vascular disease, assessed by study medical officer).

8. Current, severe psychiatric disorder (e.g., acute psychosis, severe anxiety and/or mood disorder, intent to harm self or others assessed by study medical officer and/or psychiatrist).
9. History of glaucoma, hyperthyroidism, pheochromocytoma, motor tics, vocal tics, or Tourette's syndrome.
10. Use of monoamine oxidase inhibitors in previous 14 days, or use of other medications that could interact with study medication on assessment by the investigator.
11. Exposure to any investigational drug within the 4 weeks prior to screening
12. Not available for follow-up (e.g., likely travel or imprisonment).
13. Undergoing child protection service/court/work-ordered drug testing.

3.2. INTERVENTION

Participants receive 250mg oral lisdexamfetamine/d over 12 weeks preceded by 7 days induction of 150mg/d and followed by a two week reduction period of 150mg/d for 7 days and 50mg/d for 7 days or identically matched placebo. All participants are offered 4 sessions of cognitive behavioural therapy (CBT) during the treatment period.

3.3. RANDOMISATION

Participants will be randomised in a 1:1 ratio between groups, stratified by treatment site, using an independently computer-generated randomisation schedule. The randomisation schedule used blocks of size 4 and 6 with the size of block chosen at random and treatment allocations randomly permuted and balanced with blocks. The randomisation scheme was prepared using SAS 9.4.

3.4. BLINDING

Participants, treating clinicians and study personnel are blinded to study treatment allocation. The active drug (LDX dimesylate) and the placebo were provided in identical capsules

Unblinding could occur during the conduct of the trial in emergency situations where management of an participant required knowledge of treatment allocation. In the event unblinding was required, the Principal Investigator would be contacted in all these instances but kept blinded to treatment allocation.

3.5. STUDY ENDPOINTS

3.5.1. Primary endpoint

The primary endpoint is the change from baseline to week 13 in the self-reported number of days of methamphetamine use for the previous 28 days.

3.5.2. Secondary endpoints

The secondary endpoints are:

- total number of days of self-report MA use over the 12-week active treatment period (weeks 2–13);
- longest period of abstinence during the treatment period;
- percentage of participants achieving ≥ 21 consecutive days abstinence during the treatment period;
- number of MA negative UDS in the 4 weeks prior to week 13 (Treatment Effectiveness Score [2], with missing UDS assigned as positive);
- retention rate at weeks 5, 9, 13 and 19;
- measures of amphetamine craving, withdrawal, and severity of dependence:
    - Visual Analogue Scale for cravings (VAS),
    - Amphetamine withdrawal questionnaire (AWQ),
    - Severity of Dependence Scale (SDS);
- measures of abuse liability, other drug use, risk behaviour and crime:
    - Drug Evaluation Questionnaire-5 (DEQ-5),
    - Timeline follow back (all drugs—past 28 days),
    - Urine drug screen (UDS),
    - Opiate Treatment Index-Injecting Practices (OTI-I),
    - Blood borne virus transmission risk behaviour measured by days of injecting,
    - OTI-Crime (OTI-C),
    - Substance Use and Sex Index (SUSI);
- measures of physical and mental health and cognitive and psychosocial functioning:
    - Patient Health Questionnaire-15 (PHQ-15),
    - Short Form-12 Health Survey (SF-12),

    - Brief Psychiatric Rating Scale (BPRS-4),
    - Depression & Anxiety Survey Scale (DASS-21),
    - WHO Quality of Life-BREF,
    - Insomnia Severity Index (ISI);
- treatment satisfaction, measured by Treatment Satisfaction Questionnaire for Medication (TSQM);
- medication adherence:
    - supervised doses,
    - take away dispenses;
- testing the blind.

3.5.3. Safety endpoint

The safety endpoints are:

- all AEs and that are possibly or probably treatment related, that occur during the study treatment period or within 30 days of the last dose of study treatment;
- all serious adverse events (SAEs);
- discontinuations due to Treatment Emergent Adverse Events (TEAEs);
- suspected unexpected serious adverse reactions (SUSARs).

3.6. SAMPLE SIZE

Based on the primary outcome measure (days of methamphetamine use out of 28 days), to detect a mean difference of 4.5 days (9 days in control vs. 4.5 days in the LDX group out of 28 days) at week 13 (12 weeks post treatment), assuming a pooled standard deviation (SD) of 9 days (i.e. median effect size d=0.5), with over 80% power at a two-sided significance level of 0.05, a sample size of 126 (63 per group) is required. Taking into account an estimated attrition rate of 30%, 180 participants in total (90 per group) need to be randomised.

Prior to unblinding, the observed pooled standard deviation, achieved sample size and attrition rate will be used to recalculate the power of the study to detect the same effect size.

3.7. INDEPENDENT DATA SAFETY MONITORING COMMITTEE (IDSMC)

The IDSMC monitored recruitment and the data related to outcomes and safety at least annually during conduct of the study. The IDSMC was scheduled to review unblinded data to examine participant characteristics, treatment compliance, outcomes and adverse events, when the 50th participant completed treatment or 6 months after the commencement of recruitment, whichever occurred first, with subsequent meetings in accordance with the IDSMC charter. The DSMB could recommend stopping the trial if the number and/or severity of adverse events justified discontinuation of the study; or on the basis of a positive efficacy result only when the primary endpoint (methamphetamine use) data are truly compelling and the risk of a false positive conclusion was acceptably low ($p<0.001$ for primary endpoint). The DSMB met on the 30th May 2019, 12th February 2020, and 7th October 2021, and recommended continuation of the trial each time.

3.8. DATA COLLECTION AND SECURITY

This study uses electronic data capture in the form of REDCap (Research Electronic Data Capture). The REDCap database is located on a standalone database server hosted by Hunter New England Local Health District (HNELHD). Backup systems and firewalls are in place. Access to the system is restricted to authorised personnel and password protected.

All data entered into REDCap have supporting identifiable source documentation, which are held confidentially in line with current legislation governing health information, and destroyed 15 years after study completion.

4. STATISTICAL METHODS

4.1. GENERAL CONSIDERATIONS

4.1.1. Analysis principles

Summaries of continuous variables will be presented as means and standard deviations, or as medians and interquartile ranges, as appropriate. Categorical variables will be presented as frequencies and percentages.

For comparisons between the two randomised groups, the estimand of interest is the expected difference in outcomes between the prescribed course of LDX and usual care, among participants who receive at least one dose. This corresponds to a modified intention-to-treat (ITT) approach to the analyses, whereby all randomised participants who received at least one dose of the prescribed medication are included according to their allocated arm. That is, withdrawal prior to receiving the first dose will be handled using a principal stratum strategy, on-study death will be handled using a hypothetical strategy, and all other intercurrent events will be handled using a treatment policy strategy.

For each outcome variable, statistical significance will be assessed at the 0.05 level using a two-sided test. In addition to treatment effect estimates and p-values, 95% confidence intervals will be reported to describe uncertainty about the effect of treatment.

No adjustment for multiple testing is planned, as the primary comparison is at a single predefined time point (week 13). However, all results will be reported and interpreted by considering both the strength of the evidence and the consistency of effects across endpoints.

All analyses will be performed using SAS version 9.3 or later, and R 4.0 or later.

4.1.2. Analysis dataset

The primary and secondary outcomes will be analysed on the full analysis set following the modified ITT principle described above; that is, by including all randomised participants who received at least one dose of the prescribed medication according to the group to which they were randomised and regardless of protocol compliance.

4.1.3. **Missing data handling**

The patterns of missing data over time and between groups will be summarised in tables and graphs. Reasons for missingness will be described and explored. Logistic regression will be used to explore the potential factors related to missingness and withdrawal. The handling of missing data will follow the National Research Council (US) Panel recommendations on Handling Missing Data in Clinical Trials [3], as described below and in Section 4.3.

For longitudinal analyses, the likelihood-based mixed model for repeated measures (MMRM) approach will be utilised, which depends on a missing at random (MAR) assumption [4]. The MMRM approach makes use of all available data and is reliable for effect and standard error estimates under the MAR assumption.

Sensitivity analyses will be performed to assess the robustness of the main results to different missing data assumptions [3] [5]. Details can be found in Section 4.3.3.1.

4.2. SUMMARY OF STUDY DATA

4.2.1. Protocol deviations

Protocol deviations will be summarised as the number of deviations by type (randomisation of an ineligible participant, failure to comply with study treatment, and other). All protocol deviations will be listed with a description of the deviation and the corrective action taken.

4.2.2. Participant disposition

All subjects who were invited to participate in this trial will be accounted for, and a CONSORT flow chart will be prepared. Reasons for early withdrawal will be listed for all participants that prematurely withdrew from the study. The number of participants that were screened but not randomised will be presented and the reasons for their non-participation will be listed (where available).

4.2.3. Participant characteristics

Baseline demographic variables such as age and gender, and relevant clinical variables will be summarised for each treatment group.

4.2.4. Concomitant therapy

Concomitant medications will be categorised based on the WHO Anatomical Therapeutic Chemical classification system. The frequencies and percentages of participants taking concomitant medications before and after randomisation will be summarised by treatment group.

4.2.5. Treatment adherence

Medication adherence is assessed daily for days 1–5 of week 1 and weekly thereafter. The number of CBT sessions attended (out of a total possible of four) will be recorded. These will be summarised by treatment group.

4.3. ANALYSIS OF PRIMARY OUTCOME

4.3.1. Analysis method

The primary outcome is the number of days of self-reported methamphetamine use in the past 28 days. The primary efficacy measure is the difference between the two groups in the change in the number of days of methamphetamine use from baseline to week 13.

A likelihood-based mixed effects regression model approach will be used for the primary efficacy analysis. The days of methamphetamine use at baseline and all post-baseline assessments (weeks 5, 9, 13 and 19) will be the time-varying dependent variable. Covariates will include (categorical) visit, randomised treatment group, and their interaction, with the coefficient corresponding to the difference between groups in the change from baseline to week 13 being the parameter of primary interest. Site will also be included as a covariate in order to account for its use in the stratification of the randomisation. Participant-specific random effects will be included in order to account for temporal correlation in the outcome measure.

The initial model will assume a multivariate normal distribution. However, if residual-based model checks suggest that this is inappropriate (e.g., due to heteroscedasticity), a generalised linear mixed effect models will be considered: a binomial or double binomial (in the case of evidence of under- or overdispersion) outcome distribution may be more appropriate.

4.3.2. Supplementing missing TLFB28 data with available TLFB7 data

The number of days of MA use in the past 28 days at baseline and weeks 5, 9, 13 and 19 was collected

during the study period using the 28-day timeline follow back (TLFB28) approach. At weekly visits during the study period up until week 13, the number of days of MA use in the past 7 days was also collected using the 7-day timeline follow back (TLFB7) approach.

The primary outcome measure will be based on the data collected using the TLFB28 approach. As described in Section 4.1.3, the primary analysis method (mixed effects regression) allows for missing outcome data and is valid under a missing at random (MAR) assumption. However, it is possible that there will be some 28-day periods for which the full TLFB28 outcome is missing but some TLFB7 data, covering part of that period, are available. As such, this Section describes how the available TLFB7 data will be used to supplement the missing TLFB28 data.

If at least one TLFB7 measure is available but the full 28-day outcome is not calculable and otherwise not available for a given 28-day period (i.e., the remaining TLFB7 measures are missing, along with the TLFB28 measure), multiple imputation will be employed to produce imputed 28-day outcomes based on the observed data. Specifically, imputed values for the missing TLFB7 measures will be produced using predictive mean matching based on the observed TLFB7 and TLFB28 measures, and the missing TLFB28 measures will passively imputed [6] by adding together the observed and imputed TLFB7 measures for the given 28-day period. Where a TLFB28 measure is missing and no TLFB7 measures are available for the corresponding 28-day period, the primary outcome will be left as missing.

The primary analysis method (mixed effects regression) will be applied to the imputed datasets, and the results combined according to Rubin's rules [7]. This approach allows us to use the collected 7-day measures to inform the analysis of the 28-day outcomes where they would otherwise be missing, and appropriately account for the uncertainty in doing so.

4.3.3. Supportive and sensitivity analyses

4.3.3.1. Sensitivity to missing data assumptions

As described in Sections 4.3.1 and 4.3.2, the primary analysis will use available TLFB28 data and imputed TLFB28 data for the 28-day periods where some TLFB7 data are available. This analysis is valid under a missing at random (MAR) assumption, conditional on variables included in the imputation and regression models [8]. Given the possibility of substantial missingness in the primary outcome, sensitivity analyses will be carried out to examine the robustness of the estimate from the main analysis to departures from its assumptions.

(1) sensitivity analysis assuming missing at random (MAR)

Multiple imputation by chained equation (MICE) will be performed to produce 100 complete imputed datasets [9] [10]. Variables in the MI model will include the primary outcome at baseline and weeks 5, 9, 13 and 19, covariates from the analysis model, covariates associated with discontinuation, as well as other variables and measures in the collected data.

Each imputed dataset will be analysed using the same mixed effects regression model as used for the primary analysis. The results from the imputed datasets will be pooled to form a single inference following Rubin's rules [7].

Given that the primary analysis treats the baseline outcome as a dependent variable, a second sensitivity analysis of the imputed data will be undertaken in which the baseline outcome is included as a fixed covariate, and only the post-baseline outcomes treated as dependent variables. This will evaluate the impact of the assumption in the primary analysis that the conditional distribution of the baseline outcome is the same as that of the post-baseline outcomes, which may be questionable due to the inclusion criterion that is based on the baseline days of methamphetamine use.

(2) sensitivity analysis assuming missing not at random (MNAR)

Pattern-mixture frameworks will be used to examine the robustness of the estimate from the primary analysis to deviations from the MAR assumption [11]. Specifically, controlled MI [12] will be used, in which incidental missing data (i.e., where the participant had not withdrawn from the study) will be imputed under a MAR assumption as described above, while missing data following trial withdrawal will be imputed under MNAR using both delta-based and reference-based methods.

Under the delta-based method [13], a fixed amount (δ) will be added to imputed outcomes to reflect the assumption that withdrawn participants would tend to have higher methamphetamine use than those remaining on-study. The effect of different plausible choices of δ will be assessed, both as a constant applied to all missing time points, and as a post-withdrawal slope [14].

Under the reference-based method [11], outcomes for all withdrawn participants will be imputed using observed data from the placebo group, reflecting the assumption that their outcomes will be similar to those participants not receiving active treatment ("jump to reference" imputation [11]).

Each of these imputed datasets will be analysed using the two methods described in the previous section.

Other MNAR approaches including the selection method [11] and shared parameter method through joint

modelling [15] may also be explored.

4.3.3.2. Sensitivity to baseline methamphetamine use cut-off

Prior to version 7.0 of the protocol, it was possible that participants would have at least 14 days of methamphetamine use in the 28 days prior to screening, but have a baseline TLFB28 outcome less than 14, due to a gap between screening and randomisation. These participants will be included in the primary analyses, but a sensitivity analysis will be undertaken excluding any participants whose baseline TLFB28 outcome is less than 14.

4.3.3.3. COVID-19 pandemic related modifications

Study recruitment and data collection was occurring when the COVID-19 pandemic began to affect Australia in March 2020. The impact of the pandemic on trial processes will be described according to recommendations in the CONSERVE 2021 Statement [16], and associated parameters will be summarised, including the discontinuation and withdrawal rates, medication adherence before and during the pandemic and any changes in participant demographics. The missing data patterns before and during the pandemic will also be compared.

Pandemic-related intercurrent events (e.g., missed visits due to government restrictions) are expected to affect participants in both treatment arms equally, and so for the primary estimand, these will be handled using a treatment policy strategy, as planned for non-pandemic-related intercurrent events [17].

Additional exploratory analyses will be conducted. Defining the pandemic onset date as 20 March 2020 (when recruitment to the study was temporarily suspended), participants' "pandemic status" is classified into three categories based on the timing of randomisation: (1) reached primary endpoint at week 13 before pandemic onset; (2) randomised and not yet reached primary endpoint at pandemic onset; (3) randomised after pandemic onset. We will explore the impact of adjusting the primary analysis for pandemic status, and will also assess the evidence of a difference in the treatment effect between subgroups of participants according to their pandemic status.

4.3.3.4. Supplementary analysis

In addition to the primary analysis targeting a treatment policy estimand via a modified ITT approach, we are also interested in a hypothetical "on-treatment" estimand, assessing the treatment effect if full compliance was achieved by all individuals, and a principal stratum estimand, assessing the treatment effect amongst the subset of participants who complied with the treatment regimen.

To estimate the on-treatment estimand, outcomes after stopping treatment (in either group) will be treated as missing. All such missing outcomes will be imputed using reference-based imputation [11], whereby they are assumed to follow a similar pattern as those observed in individuals who remained on treatment, according to their assigned group. Each imputed dataset will be analysed in the same way as described for the primary analysis, and the results combined using Rubin's rules [7].

To estimate the principal stratum estimand, we will undertake a "per protocol" analysis, including individuals who had at least one follow-up assessment, did not withdraw from the study for reasons unrelated to study medication, and did not have any major protocol violations. Participants will be analysed according to the treatment they actually received, ignoring any data collected after treatment withdrawal. The per protocol dataset will be analysed in the same way as described for the primary analysis.

Because of the potential for bias in the per protocol analysis due to differences between participants who would comply with each treatment [18], we may also further undertake a complier average causal effect (CACE) analysis targeting a principal stratum estimand of the treatment effect among participants who would have complied under *both* treatment arms.

4.3.3.5. Subgroup analyses

In order to assess if there is evidence of greater treatment efficacy for particular participants, we will repeat the primary analysis separately within subgroups defined by:

- Pandemic status (see Section 4.3.3.2),
- Childhood ADHD status (baseline WURS score ≥ 46) [19],
- Social support (low vs moderate/high baseline social support):
    - Low social support is defined as a score of 2 or less on at least two of items 1, 2, 3, 5 and 6 of the 7-item ESSI, and a total score of 18 or less [20].

An interaction between subgroup and treatment effect will be used to assess this question, acknowledging that this analysis will have reduced power.

4.4. ANALYSIS OF SECONDARY OUTCOMES

Secondary outcomes will be summarised within each treatment group at each time point using means and standard deviations, medians and interquartile ranges, or frequencies and percentages as

appropriate.

The secondary outcomes that are measured only once during follow-up:

- total number of days of self-report MA use,
- longest period of abstinence,
- achieving ≥ 21 days abstinence, and
- negative UDS in the 4 weeks prior to week 13,

will be analysed using a generalised linear model to estimate the effect of treatment, adjusting for site and MA use in the 28 days prior to baseline. Multiple imputation will be used to account for the uncertainty due to missing data in these outcomes.

The analyses of secondary outcomes measured longitudinally:

- measures of amphetamine craving, withdrawal, and severity of dependence,
- measures of abuse liability, other drug use, risk behaviour and crime,
- measures of physical and mental health and cognitive and psychosocial functioning,
- treatment satisfaction, and
- medication adherence

(see Section 3.5.2 for a full listing) will follow a similar approach to that described in Section 4.3 for the primary outcome: a mixed model for repeated measures that accounts for within-individual temporal correlations and includes all observations in the modified ITT dataset. For continuous or quasi-continuous outcomes (such as composite scale scores), a linear mixed model may be appropriate, but we will undertake residual checks to assess the suitability of assumptions, and use a generalised linear mixed model with an appropriate distribution for the outcome if necessary. Other types of these outcomes will be analysed with a GLMM with an appropriate distribution, again with residual checks to assess assumptions.

The study retention rate will be summarised at landmark time points (weeks 5, 9, 13 and 19) using a Kaplan–Meier estimate of time to withdrawal from baseline.

The success of blinding will be assessed by cross-tabulating participants' allocated treatment with their predicted treatment at each follow-up time point (weeks 5, 9 and 13). A Chi-squared test at each time point will indicate if there is evidence that an individual's prediction is associated with their allocated treatment.

Tertiary analyses may also be conducted, but none are planned at this stage.

4.5. ANALYSIS OF SAFETY OUTCOMES

Safety data will be analysed according to the treatment that the participants actually received. Safety analyses will include summaries of the incidence of all adverse events that are possibly or probably treatment related, that occur during the study treatment period or within 30 days of the last dose of study treatment.

All serious adverse events (SAEs) will be listed. In addition, incidence summaries similar to the ones planned for adverse events (AEs) will be performed for the SAEs. Discontinuations due to TEAEs will also be listed, and summarised by treatment group.

5. REFERENCES

[1] N. Ezard, A. Dunlop, M. Hall, R. Ali, R. McKetin, R. Bruno, N. Phung, A. Carr, J. White, B. Clifford, Z. Liu, M. Shanahan, K. Dolan, A. L. Baker and N. Lintzeris, “LiMA: A study protocol for a randomised, double-blind, placebo controlled trial of lisdexamfetamine for the treatment of methamphetamine dependence,” *BMJ Open,* vol. 8, p. e020723, 2018.

[2] W. Ling, S. Shoptaw, D. Wesson, R. A. Rawson, M. Compton and C. J. Klett, “Treatment Effectiveness Score as an Outcome Measure in Clinical Trials,” in *Medication Development for the Treatment of Cocaine Dependence: Issues in Clinical Efficacy Trials*, Rockville, MD, National Institute on Drug Abuse, 1997, pp. 208-220.

[3] National Research Council (US) Panel on Handling Missing Data in Clinical Trials, The Prevention and Treatment of Missing Data in Clinical Trials, Washington (DC): National Academies Press, 2010.

[4] M. G. Kenward, “The handling of missing data in clinical trials,” *Clinical Investigation,* vol. 3, no. 3, pp. 241-250, 2013.

[5] R. J. Little, R. D'Agostino, M. L. Cohen, K. Dickersin, S. S. Emerson, J. T. Farrar, C. Frangakis, J. W. Hogan, G. Molenberghs, S. A. Murphy, J. D. Neaton, A. Rotnitzky, D. Scharfstein, W. J. Shih, J. P. Siegel and H. Stern, “The prevention and treatment of missing data in clinical trials,” *The New England Journal of Medicine,* vol. 367, no. 14, pp. 1355-1360, 2012.

[6] I. Eekhout, H. C. de Vet, M. R. de Boer, J. W. Twisk and M. W. Heymans, “Passive imputation and parcel summaries are both valid to handle missing items in studies with many multi-item scales,” *Statistical Methods in Medical Research,* vol. 27, no. 4, pp. 1128-1140, 2018.

[7] D. B. Rubin, Multiple Imputation for Nonresponse in Surveys, John Wiley & Sons, 2004.

[8] S. Cro, T. P. Morris, M. G. Kenward and J. R. Carpenter, “Sensitivity analysis for clinical trials with missing continuous outcome data using controlled multiple imputation: A practical guide,” *Statistics in Medicine,* vol. 39, no. 21, pp. 2815-2842, 2020.

[9] S. Jolani, T. P. Debray, H. Koffijberg, S. van Buuren and K. G. Moons, “Imputation of systematically

missing predictors in an individual participant data meta-analysis: a generalized approach using MICE," *Statistics in Medicine,* vol. 34, no. 11, pp. 1841-1863, 2015.

[10] S. van Buuren, "Multiple imputation of multilevel data," in *Handbook for Advanced Multilevel Analysis*, J. J. Hox and J. K. Roberts, Eds., Routledge/Taylor & Francis Group, 2011, pp. 173-196.

[11] J. R. Carpenter, J. H. Roger and M. G. Kenward, "Analysis of longitudinal trials with protocol deviation: A framework for relevant, accessible assumptions, and inference via multiple imputation," *Journal of Biopharmaceutical Statistics,* vol. 23, no. 6, pp. 1352-1371, 2013.

[12] M. G. Kenward, "Controlled multiple imputation methods for sensitivity analyses in longitudinal clinical trials with dropout and protocol deviation," *Clinical Investigation,* vol. 5, no. 3, pp. 311-320, 2015.

[13] J. R. Carpenter and M. G. Kenward, Missing Data in Randomised Controlled Trials: A Practical Guide, Birmingham: Health Technology Assessment Methodology Programme, 2007.

[14] J. R. Carpenter and M. G. Kenward, Multiple Imputation and its Application, John Wiley & Sons, Ltd, 2013.

[15] D. Rizopoulos, Joint Models for Longitudinal and Time-to-Event Data, New York: Chapman and Hall/CRC, 2012.

[16] A. M. Orkin, P. J. Gill, D. Ghersi, L. Campbell, J. Sugarman, R. Emsley, P. G. Steg, C. Weijer, J. Simes, T. Rombey, H. C. Williams, J. Wittes, D. Moher, D. P. Richards, Y. Kasamon, K. Getz, S. Hopewell, K. Dickerson, T. Wu, A. P. Ayala, K. F. Schulz, S. Calleja, I. Boutron, J. S. Ross, R. M. Golub, K. M. Khan, C. Mulrow, N. Siegfried, J. Heber, N. Lee, P. R. Kearney, R. K. Wanyenze, A. Hróbjartsson, R. Williams, N. Bhandari, P. Jüni and A.-W. Chan, "Guidelines for reporting trial protocols and completed trials modified due to the COVID-19 pandemic and other extenuating circumstances: The CONSERVE 2021 Statement," *JAMA,* vol. 326, no. 3, pp. 257-265, 2021.

[17] R. D. Meyer, B. Ratitch, M. Wolbers, O. Marchenko, H. Quan, D. Li, C. Fletcher, X. Li, D. Wright, Y. Shentu, S. Englert, W. Shen, J. Dey, T. Liu, M. Zhou, N. Bohidar, P.-L. Zhao and M. Hale, "Statistical issues and recommendations for clinical trials conducted during the COVID-19 pandemic," *Statistics in Biopharmaceutical Research,* vol. 12, no. 4, pp. 399-411, 2020.

[18] International Conference for Harmonisation, *ICH E9 (R1) addendum on estimands and sensitivity analysis in clinical trials to the guideline on statistical principles for clinical trials,* 2019.

[19] M. F. Ward, P. H. Wender and F. W. Reimherr, “The Wender Utah Rating Scale: An aid in the retrospective diagnosis of childhood attention deficit hyperactivity disorder,” *The American Journal of Psychiatry,* vol. 150, no. 6, pp. 885-890, 1993.

[20] The ENRICHD Investigators, “Enhancing recovery in coronary heart disease (ENRICHD): baseline characteristics,” *The American Journal of Cardiology,* vol. 88, no. 3, pp. 316-322, 2001.

[21] D. O. Scharfstein and A. McDermott, “Global sensitivity analysis of clinical trials with missing patient-reported outcomes,” *Statistical Methods in Medical Research,* vol. 28, no. 5, pp. 1439-1456, 2019.
