# Supplementary material for: Lisdexamfetamine in the treatment of methamphetamine dependence: A randomised, placebo‐controlled trial
Source: Addiction. 2024 Dec 19;120(7):1345–59. doi: 10.1111/add.16730 (PMC12128569; doi:10.1111/add.16730)
Supplement: Supplementary file 2 — Data S2. Supporting information. [file ADD-120-1345-s001.docx]

**SUPPORTING MATERIAL S2: TREATMENT OF MISSING DATA**

### Missing primary outcome data

The table below summarizes the number of individuals with missing TLFB outcomes at each visit, in each group.

|  | **Missing TLFB28** | |
| --- | --- | --- |
| **Visit** | **Placebo** | **Lisdexamfetamine** |
| Baseline | 1 | 0 |
| Week 5 | 24 | 12 |
| Week 9 | 26 | 23 |
| Week 13 | 30 | 25 |
| Week 19 | 33 | 27 |

Note that 6 participants reported fewer than 14 days of methamphetamine use in the 28 days prior to their baseline (week 1) visit. In all of these cases, they met the inclusion criterion of at least 14 days of use at the time of screening (either on Timeline Follow-back (TLFB) or Substance Use History assessments). These individuals are all included in the primary analysis. Protocol Version 7 (approved 27 January 2020) added a requirement to reassess eligibility if the gap between screening and baseline exceeded 14 days.

### Supplementing missing TLFB28 data with available TLFB7 data

Days of use was collected using the TLFB method for both 28 day (TLFB28) and seven day (TLFB7) periods. The primary outcome measure was based on the data collected using the TLFB28 approach. The primary analysis method (mixed effects regression) allows for missing outcome data and is valid under a missing at random (MAR) assumption. However, it is possible that there were some 28-day periods for which the full TLFB28 outcome is missing but some TLFB7 data, covering part of that period, are available.

If at least one TLFB7 measure was available but the full 28-day outcome was not calculable and otherwise not available for a given 28-day period (i.e., the remaining TLFB7 measures are missing, along with the TLFB28 measure), multiple imputation was employed to produce imputed 28-day outcomes based on the observed data. Specifically, imputed values for the missing TLFB7 measures were produced using predictive mean matching based on the observed TLFB7 and TLFB28 measures, and the missing TLFB28 measures were passively imputed by adding together the observed and imputed TLFB7 measures for the given 28-day period.^1^ Where a TLFB28 measure was missing and no TLFB7 measures were available for the corresponding 28-day period, the primary outcome was left as missing.

The primary analysis method (mixed effects regression) was applied to the imputed datasets, and the results combined according to Rubin’s rules.^2^ There were a total of 36 missing TLFB28 observations where the participant had some available TLFB7 observations that partially covered the same four-week period.

Using the mice package in R,^3^ a predictive mean matching algorithm was implemented to produce 100 imputed datasets, using treatment allocation, site, and all available valid TLFB7 and TLFB28 assessments as predictors. Where a TLFB7 or TLFB28 was available but did not exactly cover the exact number of required days, it was treated as missing data in the imputation algorithm, but the imputed value was ignored, and the original used in its place for the final analysis.

Imputing the TLFB28 values with passive imputation as the sum of the corresponding four TLFB7 values along with the predictive mean matching algorithm for the TLFB7s, ensured that the imputed TLFB28s were consistent with the observed TLFB7s and took integer values in the range 0–28.

### Model details

The mixed model for repeated measures assumes that the number of days of methamphetamine use for individual *i* at visit *j*, denoted *Y_ij_*, is distributed as

$$Y_{ij}|\alpha_{i}\sim D(\mu_{ij},\phi)$$

for some distribution *D* with expected value *μ_ij_*, which is related to a linear predictor via a link function *g*:

$$g\left( \mu_{ij} \right)=\alpha_{i}+{(\beta}_{0}+\gamma_{s_{i}0})+(\beta_{1}\left( j \right)+\gamma_{s_{i}1}(j))+(\beta_{2}\left( \text{trt}_{i},j \right)+\gamma_{s_{i}2}\left( \text{trt}_{i},j \right))$$

where *α_i_ ∼ N(0, σ^2^_α_)* is an individual-specific random intercept, and γ_s_ is a multivariate normal site-specific random effect with zero mean and reduced-rank covariance matrix (McGillycuddy, in press). If the visits are denoted by integers 1, 2, etc, and the treatments by 0 (control) and 1 (active), for identifiability, we fix *β_1_(1)=0* and *β_2_(0,j)=0* for all *j*. *β_2_(1,1)=0* was also fixed, which enforces the fact that the expected outcome at baseline is the same for both treatment groups — this is the constrained longitudinal data analysis (cLDA) model.^4^

The primary parameter of interest is *β_2_(1,4)*: the estimated difference in transformed means between lisdexamfetamine and placebo groups at the Week 13 visit at the ‘average’ site. We note that this model differs from that described in the Statistical Analysis Plan (Supporting material S1), which included a single term for site as a fixed effect, rather than as a multivariate random effect, as requested by the statistical review of this manuscript.

The primary analysis initially assumed multivariate normality of the outcome (*D=N*), but diagnostic plots identified that this assumption was inappropriate, and we found that a beta-binomial distribution appeared to fit the data well. This is described in the following section.

### Model diagnostics

In order to make this modelling decision prior to unblinding, this process — including the imputation of TLFB7 described previously — was undertaken with the participants assigned to dummy allocations. For the final analysis, the imputation re-done using the true allocations.

For each imputed dataset, the proposed model was fitted using the glmmTMB package, ^5^and used the DHARMa package to simulate 1000 probability integral transform residuals.^6^ Under an appropriate model these should be approximately uniformly distributed, so we can use their distribution as a diagnostic for the suitability of the outcome distribution.

***Normal distribution***

In order to include TLFB observations that did not cover exactly 28 days, the outcome was treated as a proportion. That is:

$$Y_{ij}^{*}={Y_{ij}}/{d_{ij}}$$

where *d_ij_* is the number of days that the TLFB covered, and

$$Y_{ij}^{*}|\alpha_{i}\sim N(\mu_{ij},\sigma^{2})$$

The link function is the identity function, so *β_2_(1,j)* is an estimate of the difference in the proportion of days of use between the groups. This can be multiplied by 28 to obtain the expected difference in TLFB28 responses.

The following graph shows a uniform Q–Q plot of the residuals from the normal linear mixed model for the original data and the first five imputed datasets. If the distribution fits the data well, the points should lie on the 1:1 line.

**
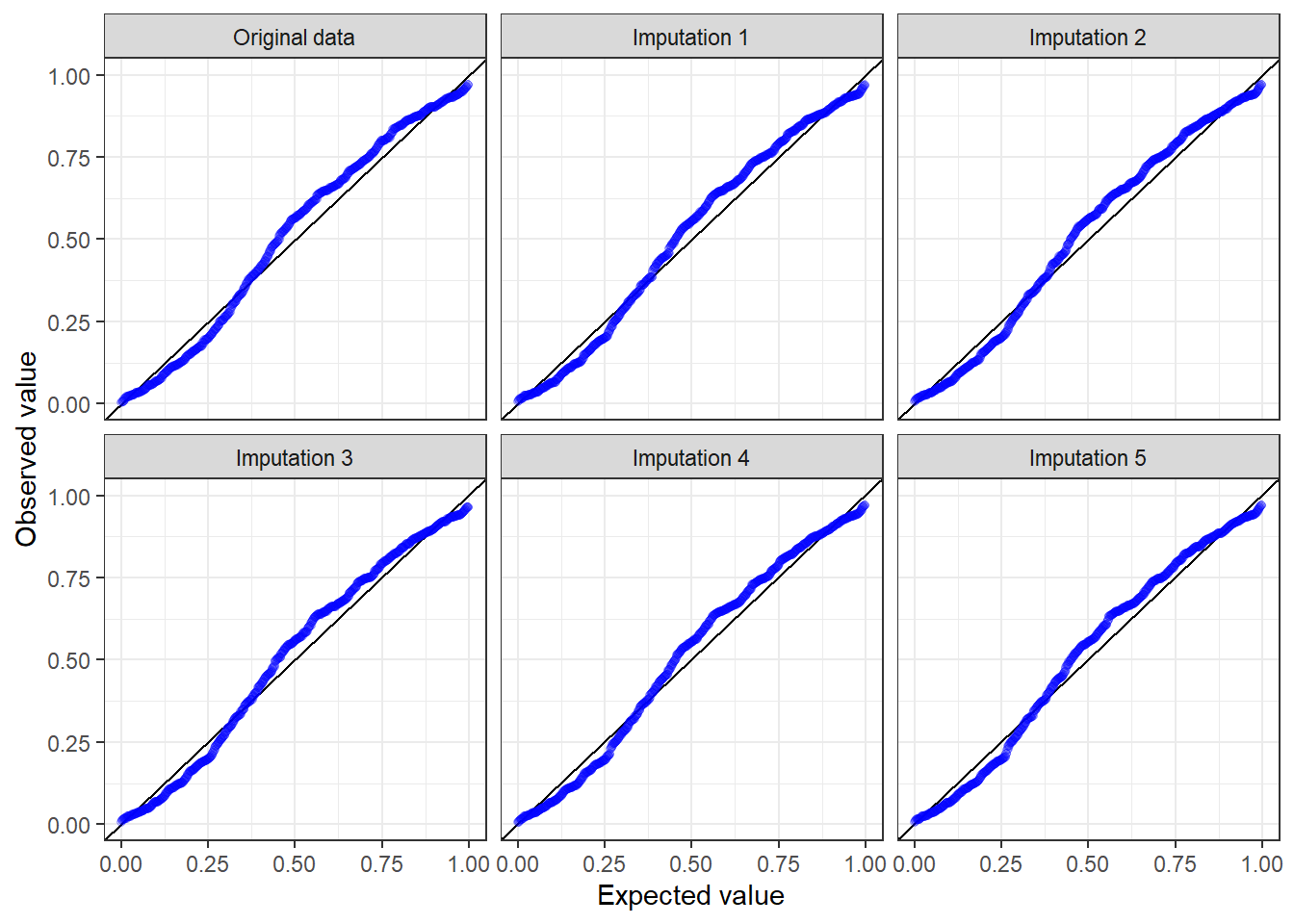
**

There was a clear deviation from the expected distribution, so the normal model did not seem appropriate for this data.

**Binomial distribution**

Under a binomial generalized linear mixed model,

$$Y_{ij}|\alpha_{i}\sim Bin(d_{ij},\pi_{ij})$$

where *d_ij_* is the number of days covered by the TLFB, and *π_ij_* is the probability of reporting methamphetamine use on each day. The link function is the logit:

$$\text{logit}\left( \pi\right)= \log\left( \frac{\pi}{1-\pi} \right)$$

such that *β_2_(1,j)* is an estimate of the log odds ratio for reporting methamphetamine use between the two groups.

The following graph shows a uniform Q–Q plot of the residuals from the binomial model for the original data and the first five imputed datasets.

**
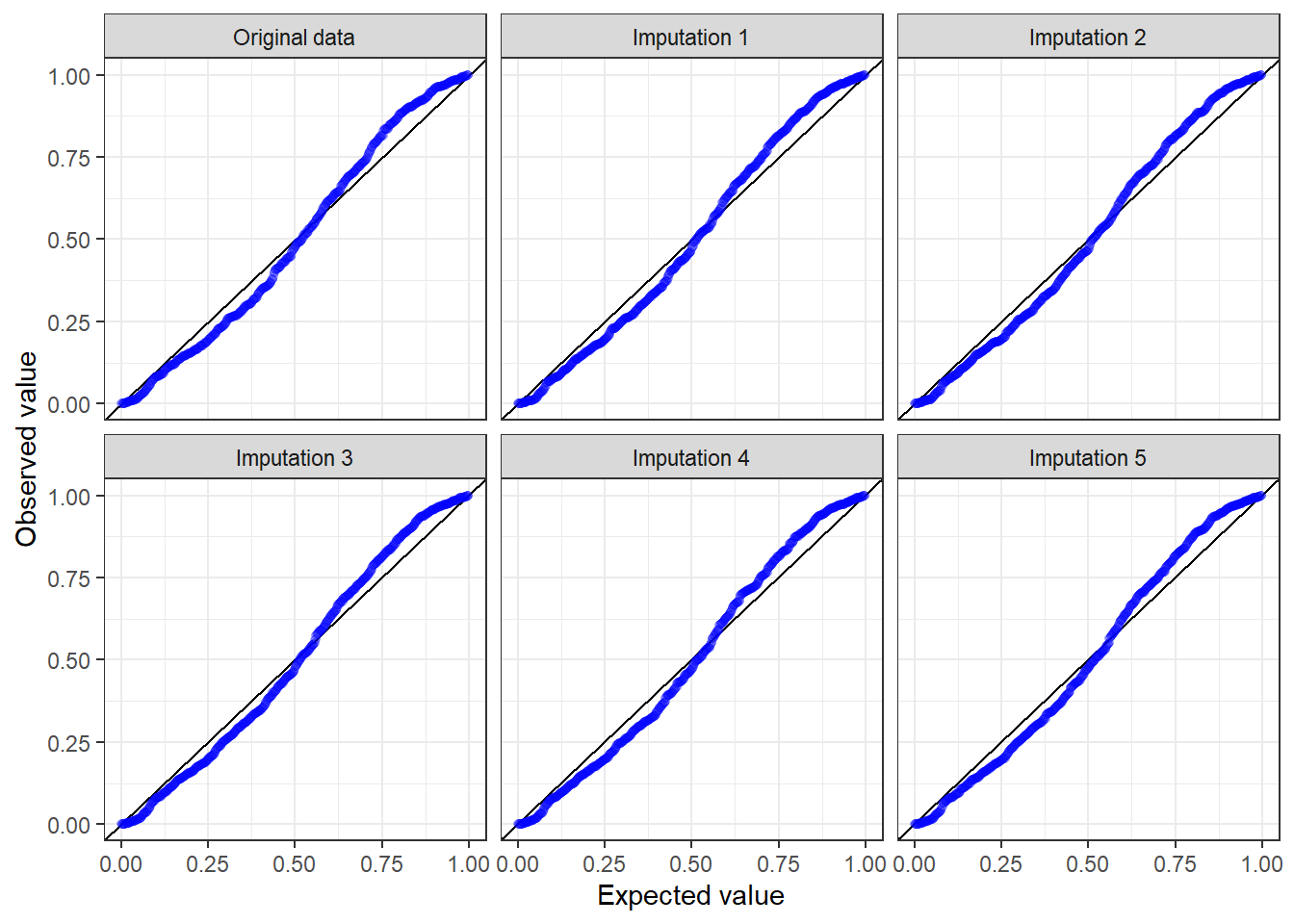
**

There is a clear deviation from the expected distribution. Further diagnostics were undertaken to examine this.

Under the binomial model, the variance of the outcome is a function of the probability of methamphetamine use:

$$Var\left( Y_{ij} | \alpha_{i} \right)=d_{ij}\pi_{ij}(1-\pi_{ij})$$

The following plots show the results of simulation-based dispersion tests which compare the variance of the observed raw residuals against the variance of the simulated residuals.

**
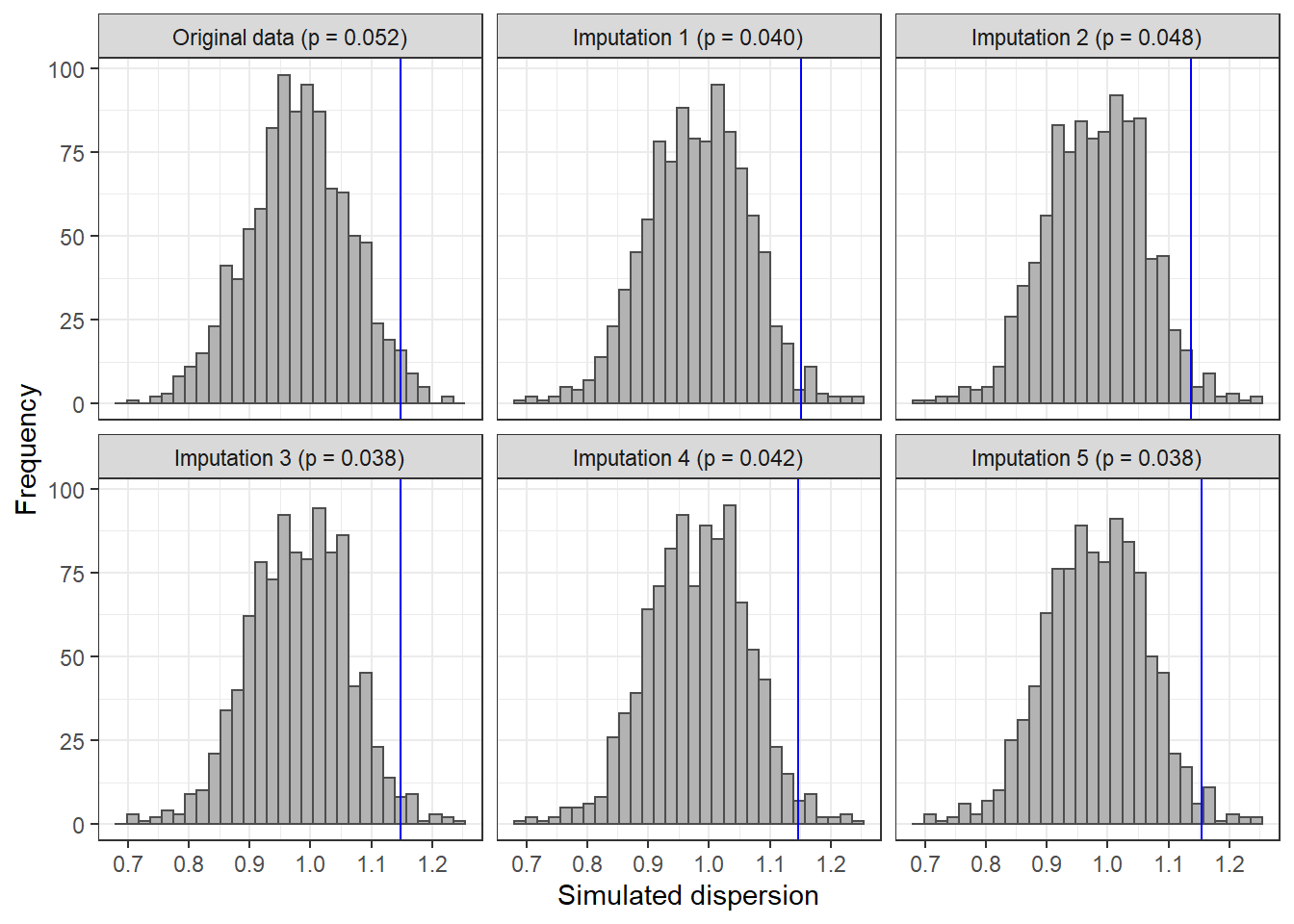
**

These show that the observed dispersion (the blue line) was consistently higher than the bulk of the distribution of simulated dispersion values, suggesting that the observed data were overdispersed relative to the binomial distribution.

***Beta-binomial distribution***

The beta-binomial distribution has an additional parameter *ϕ* that allows the possibility for overdispersion relative to the binomial:

$$Var\left( Y_{ij} | \alpha_{i} \right)=d_{ij}\pi_{ij}(1-\pi_{ij})\frac{\phi+d_{ij}}{\phi+1}$$

When *ϕ* is very large, the overdispersion factor approaches 1 and so the distribution approaches the binomial.

As with the binomial model, the link function is the logit, such that *β_2_(1,j)* is an estimate of the log odds ratio for reporting methamphetamine use between the two groups.

The uniform Q–Q plot of the residuals from the beta-binomial model for the original data and the first five imputed datasets appear to be a better fit:

**
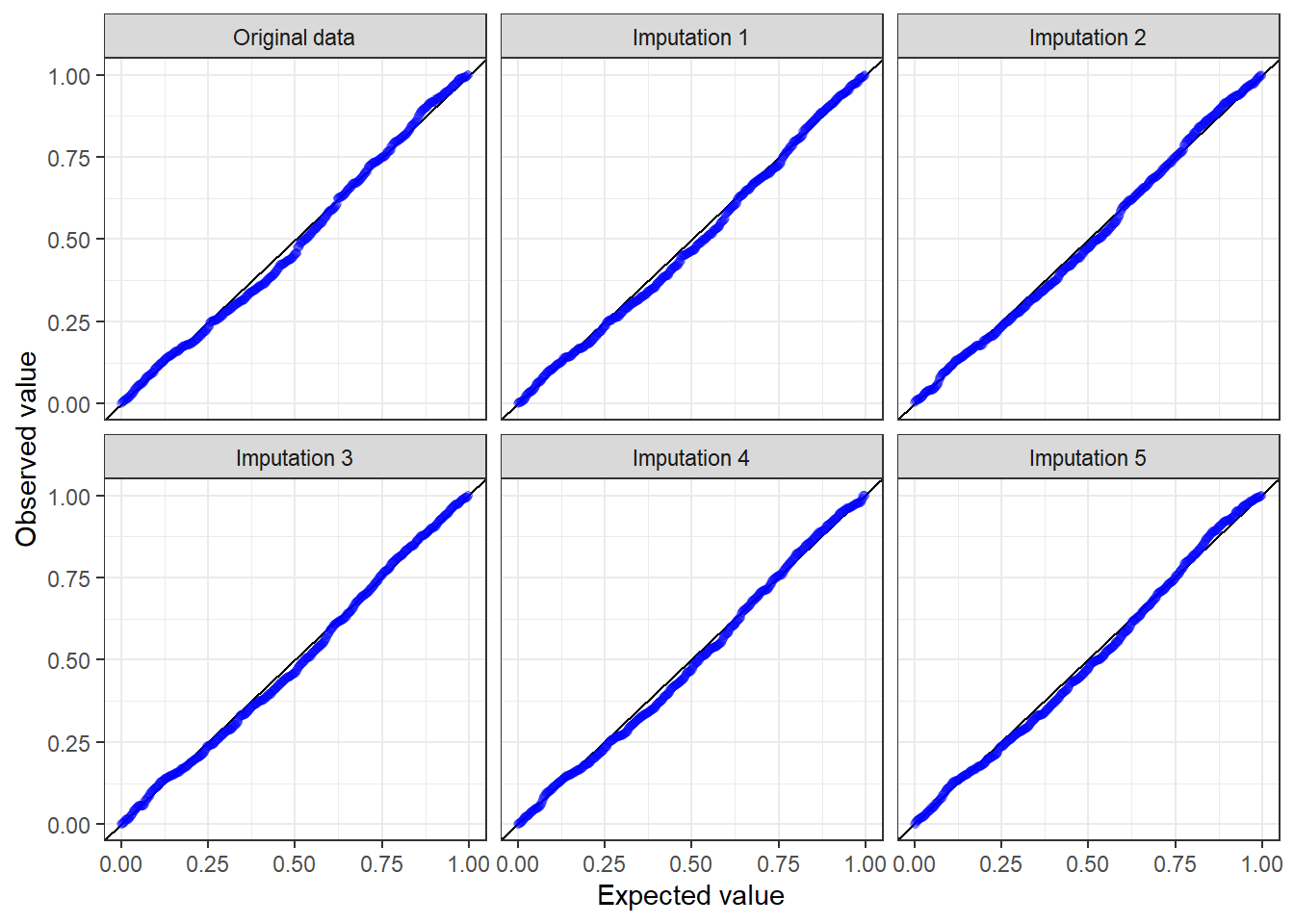
**

and the observed dispersion was consistent with the simulated values:

**
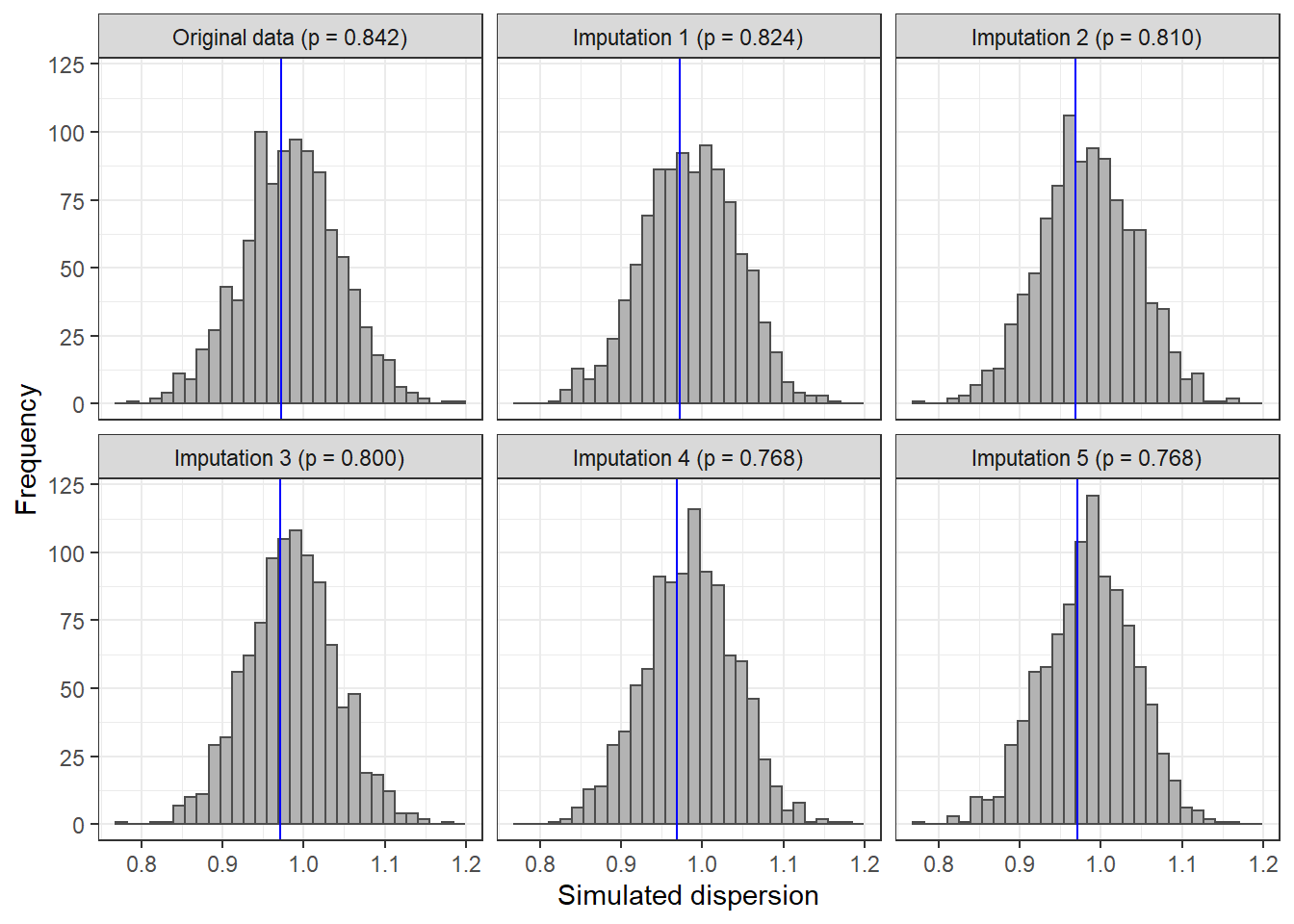
**

### Results from primary analysis

Having tested the model fit on dummy allocations, the beta-binomial model was used for the primary analysis. The model was fitted to each of the 100 imputed datasets, and combined the results using Rubin’s rules.^2^

The following table shows the estimated odds ratio (with 95% confidence interval) for the difference between the groups in the expected (daily) probability of methamphetamine use in the 28 days prior to each post-baseline visit. An odds ratio lower than 1 indicates a lower expected rate of methamphetamine use in the lisdexamfetamine compared to the placebo group at the given visit.

| **Visit** | **Odds ratio (95% CI)** | **p-value** |
| --- | --- | --- |
| Week 5 | 0.56 (0.33, 0.94) | 0.029 |
| Week 9 | 0.52 (0.28, 0.97) | 0.040 |
| Week 13 | 0.72 (0.29, 1.82) | 0.489 |
| Week 19 | 1.26 (0.47, 3.39) | 0.649 |

Since these odds ratios were on a multiplicative scale, the difference in the number of days of methamphetamine use between the groups at each time point cannot be directly estimated.

In order to estimate this difference, the fitted number of days of methamphetamine use for each individual at each visit under both allocations was obtained, and the average across the cohort was calculated. We used the delta method to estimate the variance of these estimates and hence obtain confidence intervals and p-values.

Taking the predict-then-combine approach, this estimation was done within each imputed dataset, and the results pooled using Rubin’s rules.^2^

The following table shows the estimated average number of days of methamphetamine use in each group in the 28 days prior to each visit, and the difference between the groups, along with their 95% confidence intervals. Note that the difference at Baseline is fixed to be zero by the cLDA model.

| **Visit** | **Placebo** | **Lisdexamfetamine** | **Difference** |
| --- | --- | --- | --- |
| Baseline | 23.8 (23.1, 24.4) | 23.8 (23.1, 24.4) | 0.0 |
| Week 5 | 18.8 (17.4, 20.3) | 15.8 (14.2, 17.4) | -3.0 (-5.5, -0.6) |
| Week 9 | 17.8 (16.1, 19.5) | 14.2 (12.5, 16.0) | -3.6 (-6.2, -0.9) |
| Week 13 | 16.0 (14.2, 17.8) | 13.8 (12.0, 15.6) | -2.2 (-5.0, 0.5) |
| Week 19 | 15.0 (13.1, 17.0) | 15.6 (13.6, 17.5) | 0.5 (-2.5, 3.5) |

## Sensitivity analysis assuming missing-at-random (MAR)

The primary analysis of the primary outcome is a mixed model for repeated measures (MMRM) based on a beta-binomial distribution for the number of days of methamphetamine use, conditional on site, time point, treatment allocation and an individual-specific random effect. This is valid under a missing-at-random (MAR) assumption, such that the probability of missingness is dependent only on observed covariates.

In this section two alternative methods of analysis that are also valid under a MAR assumption are considered. Both require that the missing primary outcome data are imputed. As specified in the Statistical Analysis Plan, the imputation model included the primary outcome and covariates associated with discontinuation.

To determine a set of covariates associated with discontinuation, backward elimination was used based on Bayesian information criterion in a proportional odds regression model for the number of missed visits. The analysis model included site, treatment allocation, and baseline methamphetamine use; these were forced into the discontinuation regression model. The additional candidate variables entered at the first step were:

- Gender identity
- Age
- Age of first methamphetamine use
- Baseline injecting drug use
- Baseline WURS score
- Education (completed high school)

After performing backward elimination, the variables remaining were: site, treatment allocation, baseline methamphetamine use, and age.

The following table presents the results from the primary analysis method (Model A) applied to the imputed data:

| Visit | Odds ratio (95% CI) | Diff. in days of use (95% CI) | p-value |
| --- | --- | --- | --- |
| Week 5 | 0.59 (0.35, 0.99) | -2.7 (-5.1, -0.3) | 0.047 |
| Week 9 | 0.56 (0.31, 1.02) | -3.0 (-5.5, -0.5) | 0.057 |
| Week 13 | 0.74 (0.36, 1.54) | -1.8 (-4.3, 0.8) | 0.424 |
| Week 19 | 1.26 (0.52, 3.03) | 0.8 (-2.5, 4.0) | 0.609 |

An alternative analysis (Model B) was also performed, in which baseline methamphetamine use was included as a covariate rather than treated as an outcome. The results from this analysis applied to the imputed data are presented in the table below:

| Visit | Odds ratio (95% CI) | Diff. in days of use (95% CI) | p-value |
| --- | --- | --- | --- |
| Week 5 | 0.59 (0.32, 1.08) | -2.4 (-5.2, 0.4) | 0.086 |
| Week 9 | 0.56 (0.29, 1.07) | -2.6 (-5.5, 0.2) | 0.078 |
| Week 13 | 0.73 (0.35, 1.52) | -1.4 (-4.3, 1.6) | 0.402 |
| Week 19 | 1.26 (0.50, 3.18) | 1.1 (-2.4, 4.7) | 0.625 |

Comparing the results from these three analyses for the estimated difference in total days of methamphetamine use (up to Week 13):

| Model / scenario | Diff. in total days of use (95% CI) | p-value |
| --- | --- | --- |
| Primary analysis | -8.8 (-15.0, -2.7) | 0.005 |
| MAR sensitivity analysis (Model A) | -7.4 (-13.4, -1.3) | 0.017 |
| MAR sensitivity analysis (Model B) | -6.4 (-13.9, 1.1) | 0.093 |

## Sensitivity analysis assuming missing-not-at-random (MNAR)

To test the sensitivity of the primary analysis to departures from the MAR assumption, a pattern-mixture framework, implemented via controlled multiple imputation was used.

First, a delta-based method was considered, in which the imputed data (as described in the previous section) was edited by adding days of use to reflect the MNAR assumption that withdrawn participants would tend to have higher rates of methamphetamine use than otherwise similar participants who remained on-study. First, a “worst-case scenario” was considered, in which all participants with (non-incidental) missing outcome data were assumed to have used methamphetamine for all 28 days prior to the scheduled visit.

The table below contains the results from the primary analysis method (Model A) applied to the worst-case imputed data:

| **Visit** | **Odds ratio (95% CI)** | **Diff. in days of use (95% CI)** | **p-value** |
| --- | --- | --- | --- |
| Week 5 | 0.47 (0.24, 0.92) | -2.6 (-5.0, -0.2) | 0.029 |
| Week 9 | 0.51 (0.26, 0.98) | -2.3 (-4.7, 0.1) | 0.044 |
| Week 13 | 0.71 (0.36, 1.42) | -1.3 (-3.6, 1.1) | 0.336 |
| Week 19 | 1.05 (0.54, 2.04) | -0.1 (-2.4, 2.2) | 0.891 |

And this table contains the results from the alternative analysis (Model B) applied to the worst-case imputed data:

| **Visit** | **Odds ratio (95% CI)** | **Diff. in days of use (95% CI)** | **p-value** |
| --- | --- | --- | --- |
| Week 5 | 0.37 (0.13, 1.04) | -2.9 (-6.2, 0.3) | 0.059 |
| Week 9 | 0.39 (0.14, 1.08) | -2.7 (-5.9, 0.5) | 0.069 |
| Week 13 | 0.60 (0.24, 1.49) | -1.8 (-5.1, 1.4) | 0.271 |
| Week 19 | 0.94 (0.36, 2.47) | -0.6 (-3.8, 2.6) | 0.897 |

Comparing the results for the total days of methamphetamine use under these worst-case models to the primary analysis:

| **Model / scenario** | **Diff. in total days of use (95% CI)** | **p-value** |
| --- | --- | --- |
| Primary analysis | -8.8 (-15.0, -2.7) | 0.005 |
| Worst-case MNAR analysis (Model A) | -6.1 (-11.8, -0.5) | 0.033 |
| Worst-case MNAR analysis (Model B) | -7.5 (-16.4, 1.4) | 0.100 |

Secondly, a reference-based method was used: specifically “jump-to-reference” imputation in which outcomes for missed visits were imputed using only data from the placebo arm.^7^ This reflects an assumption that the outcomes for withdrawn participants will be similar to those of participants not receiving active treatment.

The table below contains the results from the primary analysis method (Model A) applied to the jump-to-reference imputed data:

| **Visit** | **Odds ratio (95% CI)** | **Diff. in days of use (95% CI)** | **p-value** |
| --- | --- | --- | --- |
| Week 5 | 0.66 (0.40, 1.09) | -2.2 (-4.7, 0.2) | 0.105 |
| Week 9 | 0.65 (0.37, 1.14) | -2.3 (-4.8, 0.1) | 0.135 |
| Week 13 | 0.85 (0.41, 1.75) | -1.2 (-3.7, 1.3) | 0.652 |
| Week 19 | 1.20 (0.52, 2.75) | 0.7 (-2.4, 3.7) | 0.669 |

And this table contains the results from the alternative analysis (Model B) applied to the jump-to-reference imputed data:

| **Visit** | **Odds ratio (95% CI)** | **Diff. in days of use (95% CI)** | **p-value** |
| --- | --- | --- | --- |
| Week 5 | 0.65 (0.37, 1.13) | -2.1 (-4.8, 0.6) | 0.128 |
| Week 9 | 0.65 (0.36, 1.16) | -2.1 (-4.9, 0.6) | 0.146 |
| Week 13 | 0.83 (0.41, 1.69) | -0.9 (-3.7, 1.9) | 0.612 |
| Week 19 | 1.18 (0.50, 2.76) | 0.9 (-2.4, 4.2) | 0.703 |

Comparing the results for the total days of methamphetamine use under these jump-to-reference models to the primary analysis:

| **Model / scenario** | **Diff. in total days of use (95% CI)** | **p-value** |
| --- | --- | --- |
| Primary analysis | -8.8 (-15.0, -2.7) | 0.005 |
| Jump-to-reference MNAR analysis (Model A) | -5.8 (-11.6, 0.1) | 0.053 |
| Jump-to-reference MNAR analysis (Model B) | -5.1 (-12.1, 1.8) | 0.147 |

Third, as suggested by a reviewer, a “return-to-baseline” imputation was used. Here, outcomes for missed visits were imputed as the past 28-day methamphetamine use that the participant reported at baseline.

The table below contains the results from the primary analysis method (Model A) applied to the return-to-baseline imputed data:

| **Visit** | **Odds ratio (95% CI)** | **Diff. in days of use (95% CI)** | **p-value** |
| --- | --- | --- | --- |
| Week 5 | 0.51 (0.30, 0.86) | -3.1 (-5.4, -0.9) | 0.013 |
| Week 9 | 0.52 (0.31, 0.87) | -3.0 (-5.3, -0.8) | 0.013 |
| Week 13 | 0.68 (0.36, 1.27) | -2.2 (-4.4, 0.1) | 0.225 |
| Week 19 | 0.93 (0.52, 1.68) | -0.7 (-2.9, 1.6) | 0.815 |

And this table contains the results from the alternative analysis (Model B) applied to the return-to-baseline imputed data:

| **Visit** | **Odds ratio (95% CI)** | **Diff. in days of use (95% CI)** | **p-value** |
| --- | --- | --- | --- |
| Week 5 | 0.46 (0.23, 0.92) | -3.4 (-6.0, -0.7) | 0.028 |
| Week 9 | 0.47 (0.24, 0.91) | -3.3 (-6.0, -0.6) | 0.026 |
| Week 13 | 0.60 (0.30, 1.17) | -2.4 (-5.0, 0.3) | 0.134 |
| Week 19 | 0.85 (0.43, 1.69) | -0.9 (-3.6, 1.8) | 0.639 |

Comparing the results for the total days of methamphetamine use under these return-to-baseline models to the primary analysis:

| **Model / scenario** | **Diff. in total days of use (95% CI)** | **p-value** |
| --- | --- | --- |
| Primary analysis | -8.8 (-15.0, -2.7) | 0.005 |
| Return-to-baseline MNAR analysis (Model A) | -8.3 (-13.8, -2.9) | 0.003 |
| Return-to-baseline MNAR analysis (Model B) | -9.0 (-16.1, -2.0) | 0.012 |

**References**

1. Eekhout I, de Vet HCW, de Boer MR, Twisk JWR, Heymans MW. Passive Imputation and Parcel Summaries Are Both Valid to Handle Missing Items in Studies with Many Multi-Item Scales. Stat Methods Med Res*.* 2018;27(4):1128–40. doi:10.1177/0962280216654511.

2. Rubin JB. Multiple Imputation for Nonresponse in Surveys. Wiley-Interscience, Hoboken; 2004

3. van Buuren S, Groothuis-Oudshoorn K. mice: Multivariate Imputation by Chained Equations in r. J Stat Softw. 2011;45(3):1–67. doi:10.18637/jss.v045.i03.

4. Liang KY, Zeger SL. Longitudinal Data Analysis of Continuous and Discrete Responses for Pre-Post Designs. Sankhya Ser B. 2000;62(1):134–48. doi:10.2307/25053123

5. Brooks ME, Kristensen K, van Benthem KJ, Magnusson A, Berg CW, Nielsen A, Skaug HJ, Maechler M, Bolker BM. glmmTMB Balances Speed and Flexibility Among Packages for Zero-Inflated Generalized Linear Mixed Modeling. R J. 2017;9(2): 378–400. https://journal.r-project.org/archive/2017/RJ-2017-066/index.html

6. Hartig F. DHARMa: Residual Diagnostics for Hierarchical (Multi-Level / Mixed) Regression Models. 2022. https://CRAN.R-project.org/package=DHARMa.

7. Carpenter JR, Roger JH, Kenward MG. Analysis of Longitudinal Trials with Protocol Deviation: A Framework for Relevant, Accessible Assumptions, and Inference via Multiple Imputation. J Biopharm Stat. 2013;23(6):1352–71. doi:10.1080/10543406.2013.834911
